# Peptide discovery across the spectrum of neuroinflammation; microglia and astrocyte phenotypical targeting, mediation, and mechanistic understanding

**DOI:** 10.3389/fnmol.2024.1443985

**Published:** 2024-11-20

**Authors:** Benjamin A. Benita, Kyle M. Koss

**Affiliations:** ^1^Department of Surgery, University of Arizona, Tucson, AZ, United States; ^2^Department of Neurobiology, University of Texas Medical Branch (UTMB) at Galvestion, Galvestion, TX, United States; ^3^Sealy Institute for Drug Discovery (SIDD), University of Texas Medical Branch (UTMB) at Galvestion, Galvestion, TX, United States

**Keywords:** peptide, glia, microglia, astrocytes, neuroinflammation

## Abstract

Uncontrolled and chronic inflammatory states in the Central Nervous System (CNS) are the hallmark of neurodegenerative pathology and every injury or stroke-related insult. The key mediators of these neuroinflammatory states are glial cells known as microglia, the resident immune cell at the core of the inflammatory event, and astroglia, which encapsulate inflammatory insults in proteoglycan-rich scar tissue. Since the majority of neuroinflammation is exclusively based on the responses of said glia, their phenotypes have been identified to be on an inflammatory spectrum encompassing developmental, homeostatic, and reparative behaviors as opposed to their ability to affect devastating cell death cascades and scar tissue formation. Recently, research groups have focused on peptide discovery to identify these phenotypes, find novel mechanisms, and mediate or re-engineer their actions. Peptides retain the diverse function of proteins but significantly reduce the activity dependence on delicate 3D structures. Several peptides targeting unique phenotypes of microglia and astroglia have been identified, along with several capable of mediating deleterious behaviors or promoting beneficial outcomes in the context of neuroinflammation. A comprehensive review of the peptides unique to microglia and astroglia will be provided along with their primary discovery methodologies, including top-down approaches using known biomolecules and naïve strategies using peptide and phage libraries.

## 1 Introduction

**Figure 1:**
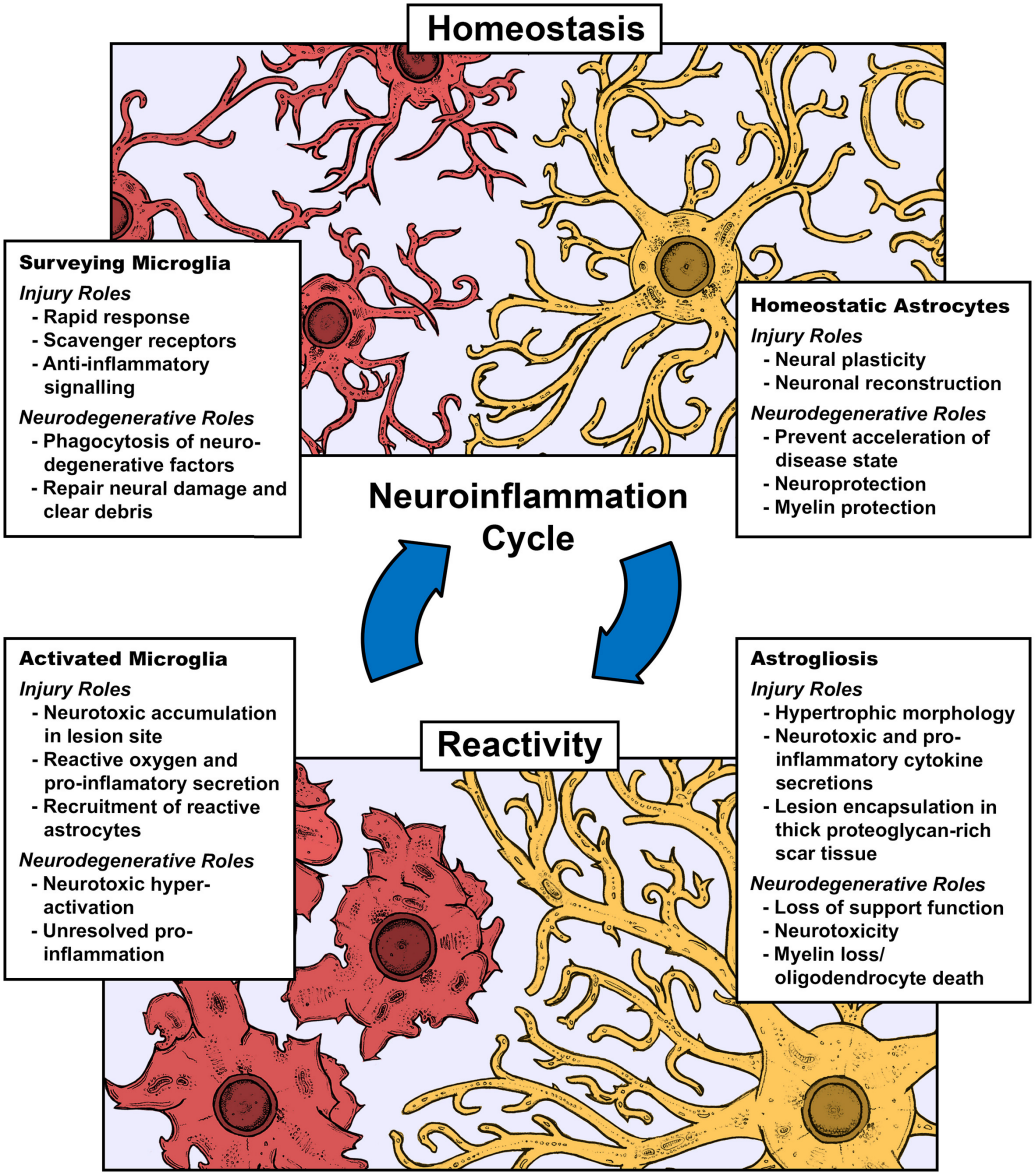
Role of microglia and astrocytes in the cycle of neuroinflammation defining key aspects of their roles under homeostasis, injury, and neurodegenerative prgression.

Neuroinflammation, inflammation centering in the central nervous system (CNS), is the core biological feature of every neural pathology be it insult or injury, including spinal cord or traumatic brain injury (SCI/TBI), and neurodegenerative disorder, including but not limited to Alzheimer's disease (AD), Parkinson's disease (PD), Huntington's disease (HD), amyotrophic lateral sclerosis (ALS), and multiple sclerosis (MS). The key arbitrators in these inflammatory events are neuroglia, known as microglia, the singular immune cells, and astrocytes, the support cell that builds the scar tissue known as gliosis (Figure 1). Microglia are highly mobile surveyors of CNS tissues with archetypical ramified morphologies that are involved in developmental (Mehl et al., 2022; Hattori, 2023), homeostatic (Li and Barres, 2018; Mordelt and de Witte, 2023), and reparative roles (Lloyd et al., 2019). This is constrasted with their roles in cell death cascades and immunity, where they behave similarly to macrophages with M1 and M2 phenotypes, representing polar ends of the pro and anti-inflammatory spetrum (Yunna et al., 2020). Astrocytes have comparable phenotypical roles in support and maintenance (Liddelow and Barres, 2017; Liddelow et al., 2020; Garland et al., 2022), including synaptic plasticity and gliotransmission (Koyama, 2015), as well as neuro and myelin protection (Burda et al., 2016; Zhou et al., 2020). As microglia react to a potent pro-inflammatory injury/neurodegenerative event, they initiate an intense necrotic cascade and recruit astrocytes to generate a proteoglycan-rich network, or gliotic scar, effectively quarantining the cascade and blocking meaningful axonal regrowth (Gao et al., 2013). Newer phenotypes are emerging, indicative of priming due to chronic inflammation, which is considered to be associated with neuropsychiatric and neurodegenerative disorders (Perry and Holmes, 2014). This allows for the identification of microglia unique to a broad spectrum of clinical issues, such as the gut-brain axis (Huang et al., 2023), neurodegenerative reactivities (Kang et al., 2018; Prater et al., 2023), and substance abuse-related cell behavior (Lacagnina et al., 2017; Melbourne et al., 2021). Astrocytes often work in subsequent tandem with microglia being first responders, often generating shared phenotypes under inflammatory and support conditions, and as such, likely have complimentary roles in these novel phenotypes. It has become impactful to understand the unique molecular patterns of these cells under their various states toward developing disease-specific biomarkers and custom therapeutics.

Many new tools and methods are emerging to characterize and facilitate the unique changes in cellular paradigms, of which peptides are rapidly becoming popular (Wang et al., 2022). With respect to targeting and mediating microglia and astroglia in phenotypically complex neuropathologies and pathophysiologies, peptide-based approaches are valid strategies. Retaining the diverse function of their protein counterparts, small protein fragments, or peptides does not require delicate 3D structures to function accordingly. As such, they have several advantages. They are robust in production methods, yields, and storage methods. Countless chemistries are available for unique modifications technically challenging or impossible with cellular translation and post-translation, which could include lipid/glycolic additions (Kowalczyk et al., 2017; Crijns et al., 2021), branching (Tam and Zavala, 1989; Elter et al., 2024), cyclization (Chung et al., 2017), organometallic additions (Reginato and Taddei, 2002), photo-labile/sensitive groups (Glatthar and Giese, 2000; Mikkelsen et al., 2018), isotopes (Zheng et al., 2016), and non-natural amino acids (Castro et al., 2023). Several biopanning and discovery tools make use of their small sequence lengths, such as display techniques and libraries (Wu et al., 2016). Peptides typically have lower computational requirements for molecular and drug discovery simulations (Rodrigues et al., 2022). Solid phase chemistry allows precise planning and the incorporation into higher order bioengineering strategies (bioconjugates, scaffolds, and drug delivery) through “lock and key” chemistries, such as click (Li et al., 2013). Although small and short in sequence, peptides can still confer secondary structures, high specificity, and potent cellular responses.

Herein, we summarize and report these strategies and functional studies on peptides effective at interacting with neuroglia. Families of neuropeptides or hormonal peptides are known to have systemic and cell-specific responses across the entire spectrum of mammalian biology (Hook et al., 2008; Burbach, 2011). Endogenously secreted in the CNS, a large family of neuropeptides have been characterized to affect a significant functional response from microglia and astrocytes, including neuroinflammatory mediation and gliotransmission (Carniglia et al., 2016). Although a few peptides have been discovered in studies for glial biology, many glial-related peptides have been discovered in extracellular matrices (ECMs) (de Castro Brás and Frangogiannis, 2020) and growth factor sources (Sporn and Roberts, 1988), such as cell adhesion molecules and transforming growth factors. More recently, system-wide immune biology and bioengineering have become clinically meaningful, which has led to a focus on inflammatory mediating peptides (La Manna et al., 2018a). Peptides are being engineered and designed to regulate inflammatory cells, including but not limited to monocytes, T cells, and organ-specific macrophages. As microglia emulate many of the pathways of immune cells, these peptides could be an excellent source for the glial researcher's toolbox.

Given that many of these peptides have been discovered indirectly, they are expected to confer limited specificity to glia subtypes and phenotypes. Using naïve blindfolded biopanning techniques, such as phage display and peptide libraries, a few studies have yielded microglia or astrocyte-specific peptides. Bacteriophages can display randomized peptide sequences on their coat at a specified length, which generates libraries with millions in sequence diversity. Further, polar M1/M2 or activated/ramified specific peptides have been discovered for the first time (Terashima et al., 2018; Koss et al., 2023). Although these peptides may have produced little to no activity beyond binding, the potential for bio-imaging and cytometric assessment is excellent. This review discusses these strategies and systematically lists the peptide studies.

## 2 Neuropeptides

One of the most intuitive sources of glial-interacial peptides are those native to the CNS. Neuropeptides are the most extensive family of molecules involved in signaling within the CNS. The hallmark characteristics of neuropeptides are their involvement in the biosynthesis and expression of genes within neurons and their role in chemical communication via regulated storage and release from secretory pathways (Hook et al., 2008). Additionally, neuropeptides are directly involved in the modulation and mediation of various neural functions through their interactions with neural receptors (Burbach, 2011). In their review, Carniglia et al. (2016) comprehensively characterized a variety of neuropeptides and their functional relation to microglia. Below is a summary of these peptides, with an added emphasis on their function in astrocytes and additional novel peptides. All neuropeptides with known interactions with microglia, astrocytes, and their receptors are fully summarized in Table 1.

**Table 1:** Summary of neuropeptide in glial biology.

**Peptide class**	**Name**	**Sequences**	**Response**	**Cell**	**Receptor**	**References**
POMC	α-MSH	SYSMEHFRWGKPV	Inflammatory mediation	AC, MG	MC1R	Lipton et al., 1998
	CRF	SEEPPISLDLTFHLLREVLEMARAEQLAQQAH SNRKLMEII	Pro-inflammation	AC, MG	CRF1-2	Jiang et al., 2019
	UROC	DNPSLSIDLTFHLLRTLLELARTQSQRERAEQ NRIIFDSV	Anti-inflammation—TLR4	MG	CRF1/2a/2b	Pedersen et al., 2002
	TP-5	RKDVY	Anti-inflammation and cytoprotection	MG	TLR2	Peng et al., 2023
PENK	LENK	YGGFL	Neuroprotection and cytoprotection	AC, MG	DOP	Qin et al., 2005; Wang et al., 2019
	MENK	YGGFM	Pro-inflammation and anti-mitotis in tumor	AC, MG	D/MOP	Stiene-Martin and Hauser, 1990; Xu et al., 2016
	DADLE	Y_D_AGF_D_L	Cytoprotection in AC and suppress M1 (TLR4)/promote M2 MG	AC, MG	D/M1OP	Wang et al., 2019; Mali and Novotny, 2022
	DAMGO	Y_D_AG-*N*(Me)F-G-ol	Gliotransmission and pro-inflammation	AC, MG	MOP	Corkrum et al., 2019; Cuitavi et al., 2023
	DPDPE	Y_D_Pen^*^GF_D_Pen^*^	Neuroprotection from AC and anti-inflammation	AC, MG	DOP	Liang et al., 2014; Shrivastava et al., 2017
	Dermorphin DALDA	Y_D_AFGYPS Y_D_RFK	Anti-inflammation and oxidative-nitrosative stress	MG	MOP	Gadepalli et al., 2023
	Deltorphin A1 Deltorphin I Deltorphin II	Y_D_MFHLMD Y_D_AFDVVG Y_D_AFEVVG	Neuropathic cold allodynia inhibition	AC	DOP	Mika et al., 2014; Reiss et al., 2021
PDYN	Endomorphin-1 Endomorphin-2	YPWF YPFF	Anti-phagocytosis, chemotaxis, and superoxide anions	MG	MOP	Azuma et al., 2001; Lu et al., 2022
	Dynorphin A Dynorphin B	GGFLRRIRPKLKWDNQ YGGFLRRQFKVVT	Anti-inflammation and pain processing	AC, MG	KOP	Liu et al., 2020; Lee et al., 2022
	β-Endorphin	YGGFMTSEKSQTPLVTLFKNAIIKNAYKKGE	Anti-inflammation and pain mediation—IL10	MG	M/D/KOP	Mao et al., 2019; Ma et al., 2021; Belo et al., 2023
	α-Neoendorphin	YGGFLRKYPK	Pro-algesia and secretion	AC	M/D/KOP	Wahlert et al., 2013
PNOC	N/OFQ	SEFMRQYLVLSMQSSQ/FGGFTGARKSARKLANQ	Differential IL1β expression and developmental glutamate/aspartate homeostasis	AC, MG	NOP	Mallimo and Kusnecov, 2013; Meyer et al., 2017
BB	GRP	VPLPAGGGTVLTKMYPRGNHWAVGHLM	Nociceptive regulation	AC, MG	GRPR	Ji et al., 2019; Liu et al., 2023
	NMB	GNLWATGHFM	Depolarization of neuronal cells	AC	NMBR	Lee et al., 1999; Mason et al., 2002
	NMC	GNHWAVGHLM	Depolarization of neuronal cells	AC	BB2R	Mason et al., 2002
	NMU	FRVDEEFQSPFASQSRGYFLFRPRN	Pro-BDNF and neuroprotection	AC, MG	NMUR1-2	Iwai et al., 2008
Pancreatic Peptides	NPY	YPSKPDNPGEDAPAEDLARYYSALRHY INLITRQRY	Anti-inflammatory and neuroprotection	AC, MG	NPYR1-5	Ferreira et al., 2010
	Amylin	KC^*^NTATC^*^ATQRLASQELHRLQTY	Anti-neuroinflammation and amyloid toxicity	AC, MG	AMYR1-3	Fu et al., 2015, 2017; Wang E. et al., 2017; Soudy et al., 2019
Intestinal Peptides	VIP	KPRRPYTDNYTRLRKQMAVKKYLNSILN	Anti-inflammation and neuroprotection	MG	VPAC1-2	Delgado and Ganea, 2003
	PACAP-27	HSDGIFTDSYSRYRKQMAVKKYLAAVL	Pro-inflammatory and neuroprotection	AC, MG	VPAC1-2 and PAC	Avila et al., 2019
	PACAP-38	HSDGIFTDSYSRYRKQMAVKKYLAAVLGKR YKQRVKNK	Pro-inflammation, neural proliferation, and M2 mediated angiogenesis	MG	VPAC1-2 and PAC	Denes et al., 2023
	GLP-2	HADGSFSDEMNTILDNLAARDFINWLIQTKITD	Anti-inflammation and neural proliferation	AC, MG	GLP2R	Velázquez et al., 2009; Zhang et al., 2021
	Ex-4	HGEGTFTSDLSKQMEEEAVRLFIEWLKNGG PSSGAPPPS	Anti-inflammation and neuroprotection	AC, MG	GLP1R	Kim et al., 2009; Shan et al., 2019
	Uroguanylin Uroguanylin active	NDDC^*^1ELC^*^2VNVAC^*^1TGC^*^2L TDEC^*^1ELC^*^2INVAC^*^1TGC^*^2	Intracellular calcium regulation	AC	GUCY2CR	Habek et al., 2021
Statins	SST-14	AGC^*^KNFFWKTFTSC^*^	Aβ co-localization and proteolytic breakdown from IDE	AC, MG	SSTR1–5	Tundo et al., 2012; Gonzalez-Rodriguez et al., 2021
	CST-14	PC^*^KNFFWKTFSSC^*^K	Anti-inflammatory, neuroprotection, and BBB integrity	AC, MG	SSTR1-5	Castillo-González et al., 2023
	CST-29	EGAPPQQSARRDRMPC^*^RNFFWKTFSSC^*^K	Anti-inflammation and glial density	AC, MG	SSTR1-5	Serrano-Martínez et al., 2024
Kinins	SP	RPKPQQFFGLM	Pro-inflammation and MG attraction/proliferation	AC, MG	NK1R-3R	Burmeister et al., 2017
	HK-1	TGKASQFFGLM	Pro-inflammation	AC,MG	NK1R-2R	Sakai et al., 2012; Aczél et al., 2020
	CCK-8	DYMGWMDF	Anti-glial activation and calcium regulation	AC, MG	CCKBR	Müller et al., 1997; Chen et al., 2021
	Bradykinin	RPPGFSPFR	Pro-inflammation, glutamate release, and neuronal crosstalk	AC, MG	B2R	Huisman et al., 2008; Liu et al., 2009; Asraf et al., 2017
Galaninergic peptides	GALA-2-11	WTLNSAGYLL	Immune mediation	AC, MG	GALR3	Priller et al., 1998; Koller et al., 2019
	Galantide (M15)	GWTLNSAGYLLGPQQFFGLM	Mitochondrial protection, and neuroprotection	AC, MG	GALR3	Schauwecker, 2010
Adrenal peptides	CGRP	ACDTATCVTHRLAGLLSRS GGVVKNNFVPTNVGSKAF	Anti-inflammtion—TLR4 and nociceptive regulation	AC, MG	CGRPR	Consonni et al., 2011; Sun C. et al., 2021
	AM	YRQSMNNFQGLRSFGCRFGTCTVQKL AHQIYQFTDKDKDNVAPRSKISPQGY	Anti-inflammtion and neuroprotection	AC, MG	CLR and RAMP	Consonni et al., 2011; Sun L. et al., 2021
	AM2	TQAQLLRVGC^*^VLGTC^*^QVQNLS HRLWQLMGPAGRQDSAPVDPSSPHSY	Anti-inflammation and neuroprotection	MG	AM2R	Lee et al., 2023
Adipose regulators	LEP	NVIQISNDLENLR	Anti-inflammation and neuroprotection	AC, MG	LEPR/OBR	Pan et al., 2008; Ma et al., 2023
	Obestatin	FNAPFDVGIKLSGVQYQQHSQAL	Anti-apoptosis, astrogliosis, and inflammation	AC	GPR39	Mirarab et al., 2019
GHS	AG	GSS(n-octanoyl)FLSPEHQRVQQRKESKKPPAKLQPR	Anti-inflammation and glucose regulation	AC, MG	GHSR1a	Frago and Chowen, 2017; Liu et al., 2019
	GRF	YADAIFTNSYRKVLGQLSARKLLQDIMSR	Inflammatory and angiogenic regulation	MG	GHRHR	Cen et al., 2022
Other hormone peptides	OXT	C^*^YIQNC^*^PLG	Anti-inflammation, neuromodulation, gliotransmission, and plasticity	AC, MG	OXTR	Wang P. et al., 2017; Baudon et al., 2022; Selles et al., 2023
	VPN/AVP	C^*^YFQNC^*^PKG	Cell volume and inflammatory regulation	AC, MG	V1aR	Sarfaraz and Fraser, 1999; Szmydynger-Chodobska et al., 2011
	APEL	QRPRLSHKGPMPF	Inflammation regulation – TLR4	AC, MG	APJ	Zhang et al., 2019; Zhou et al., 2019

Acronym nomenclature: α-MSH, α-Melanocyte-stimulating hormone; β-MSH, β-melanocyte-stimulating hormone; AG, acyl-ghrelin/ghrelin; AM, adrenomedullin; AM2R, adrenomedullin 2 receptor; AMYR1-3, amylin receptor 1-3; Aβ, amyloid beta; APEL, apelin-13; APJ, apelin receptor; AC, astrocyte; BBB, blood brain barrier; BB, bombesin peptides; BB2R, bombesin 2 receptor; BDNF, brain-derived neurotrophic factor; CGRP, calcitonin gene-related peptide; CGRPR, calcitonin gene-related peptide receptor; CCK-,8cholecystokinin / octreotide; CCKBR, cholecystokinin B receptor; CCKR, cholecystokinin receptor; CRF, corticotropin releasing factor; CRF1-2, corticotropin releasing factor type 1-2 receptor; CST-14, cortistatin 14; DOP, delta opioid receptor; Ex-4, exendin-4; GALA-2-11, galanin; GalR3, galanin receptor 3; M15, galantide; GRP, gastrin releasing peptide; GRPR, gastrin-releasing peptide receptor; GLP-2, glucagon-like peptide; GLP1R-2R, glucagon-like peptide-1-2 receptor; GPR39, g-protein coupled receptor 39; GM-CSF, granulocyte-macrophage colony-stimulating factor; GRF, growth hormone releasing factor; GHRHR, growth-hormone-releasing hormone receptor; GHS, growth hormone secretagogues; GHSR1, growth hormone secretagogue receptor; GUCY2CR, guanylyl cyclase 2C receptors; HK-1, hemokinin; IDE, insulin-degrading enzyme; IL1β, interleukin 1 beta; IL3, interleukin 3; IL10, interleukin 10; AM2, intermedin; KOP, kappa opioid receptor; LEPR/OBR, leptin or obesity receptor; LEP, leptin peptide; LENK eu-enkephalin; M1/M2, macrophage polar phenotypes reactive/ramified; MC1R, melanocortin 1 receptor; MC4R, melanocortin 4 receptor; MENK, met-enkephalin; MG, microglia; MOP, mu opioid receptor; NKA, neurokinin A; NKB, neurokinin B; NK1R-3R, neurokinin-1-3 receptor; NMB, neuromedin B; NMBR, neuromedin B receptor; NMC, neuromedin C; NMU, neuromedin U; NMUR1-2, neuromedin U receptors type 1-2; NPY, neuropeptide Y; NPYR1-5, neuropeptide Y receptor type 1-5; N/OFQ, nociceptin/orphanin; NOP, nociceptin/orphanin receptor; OXT, oxytocin; OXTR, oxytocin 1 receptor; PACAP-27, pituitary adenylate cyclase-activating polypeptide 27; PACAP-38, pituitary adenylate cyclase-activating polypeptide 38; PAC, pituitary adenylate cyclase-activating polypeptide receptor; PNOC, prepronociceptin; PDYN, prodynorphin; PENK, proenkephalin; POMC, proopiomelanocortin; PP, pancreatic peptide; RAMP, receptor-activity modifying proteins; SST-14, somatostatin 14; SST-28, somatostatin 28; SSTR1-5, somatostatin receptors 1-5; SP, substance P; TP-5, thymopentin; TLR2, toll-like receptor 2; TLR4, toll-like receptor 4; B2R, type 2 bradykinin receptor; UROC, urocortin; VIP, vasoactive intestinal peptide; VPAC1-2, vasoactive intestinal polypeptide receptor 1-2; VPN/AVP, vasopressin; V1aR, vasopressin 1a receptor.

Peptide notations: ^*^nCylic disphulide bridges where n denotes the bridge location for multiple linkages and n is not denoted when only one bridge. _D_ denotes the D stereogenic representation of the subsequent amino acid. *N*(Me) depicts an N-methyl group on a F group and G-ol refers to glycinol (ethanol substitution with ethanol). n-octanoyl is a 8 group hydrocarbon on a branched amide bond. CH_2_OH is hydroxymethyl group on the C-terminus. Pen is defined as penicillamine which also capable of forming cyclic disulphide bridges.

### 2.1 Pro-opiomelanocortin peptides

Pro-opiomelanocortin (POMC) peptides are a family of precursor proteins with various functions derived from anterior and intermediate lobes of the pituitary and some neurons in the arcuate nucleus within the hypothalamus (Harno et al., 2018). After post-translational cleavage, its derivatives include peptide hormones known as melanocortins, such as Adrenocorticotropin or adrenocorticotropic hormone (ACTH), α, β, γ-Melanocyte-Stimulating Hormones (MSH), β & γ Lipotropin, and β-Endorphins (Cawley et al., 2016). These peptides have diverse functions, including involvement in caloric intake and energy expenditure, learning and memory, and sexual behaviors (Wikberg et al., 2000). Additionally, melanocortins are tightly associated with inflammatory processes on immune cells such as macrophages, monocytes, and lymphocytes in both the central and peripheral nervous systems (Catania et al., 2004). Although the involvement of melanocortins with immune cells has been characterized, their effects on microglia are not widely understood, especially in the context of β and γ-MSH and β-Endorphins (Carniglia et al., 2017).

Bettenworth et al. used an α-MSH C-terminus fragment tripeptide K(d)PT in their in *vitro/in vivo* interleukin 10 (IL10) deficient colitis mice models to note a reduced severity in inflammation and improved transepithelial electrical resistance after interferon-gamma (IFNγ) and tumor necrosis factor-alpha (TNFα) activation (Bettenworth et al., 2011). α-MSH peptides also have a profound effect on microglia through the inhibition of toll-like receptors 2 and 4 (TLR2 and TLR4) (Carniglia et al., 2016) and the attenuation of amyloid beta (Aβ) activation (nitric oxides, TNFα, and IL6) (Galimberti et al., 1999; Lindberg et al., 2005). α-MSH has been shown to inhibit the CNS production of TNFα in a mouse-based model of neuroinflammation after lipopolysaccharide (LPS) was injected (Rajora et al., 1997). Setmelanotide, a novel melanocortin-4 receptor (MC4R) agonist, was shown to attenuate a reactive astrocyte phenotype in an *in-vitro* study on active MS lesions through the induction of IL6 and IL11. MC4R is abundant throughout the CNS, especially within astrocytes (Kamermans et al., 2019). Hippocampal rat astrocytes were found to have doubled vascular endothelial growth factor (VEGF) expression 1 h after exposure to α-MSH, which is suggestive of neuroprotective qualities of the peptide related to the subsequent release of neurotrophic growth factors (Dubynina et al., 2009). α-MSH has also been shown to be neuroprotective of neurons and glial cells through a PPAR-γ and brain-derived neurotrophic factor (BDNF) mediated mechanism in the striatum of rats exposed to 3-nitropropionic acid (3-NP), which induces the degeneration of the striatum (Saba et al., 2019). In murine microglia, αMSH reduced TNFα, nitric oxide, and IL6 production and reduced TNFα in human astrocytes (Lipton et al., 1998).

ACTH is a pituitary hormone stimulated by neuropeptides, which bind to specific G-protein coupled receptors (GPCRs), or corticotropin-releasing hormone receptors, and are crucial in regulating cortisol and stress (Lim and Khoo, 2000). Neuropeptides in this class include corticotrophin-releasing factor (CRF) and Urocortin I (UROC), which have a more complex effect on glial-mediated inflammation. CRF can activate both microglia and astrocytes related to neuropsychiatric depression (Jiang et al., 2019), while UROC has an inflammatory-mediated neuroprotective effect on hippocampal neurons (Pedersen et al., 2002). Thymopentin (TP-5) is a small peptide secreted by the thymic system known for its immunomodulatory effects on T cells (Fan et al., 2006). It can also enhance levels of ACTHs and endorphins (Malaise et al., 1987). TP-5 is capable of inhibiting LPS reactive microglia in the nod-like receptor protein 3 (NLRP3) and nuclear factor kappa B (NFκB) pathways, facilitating a neuroprotective outcome (Peng et al., 2023).

### 2.2 Opioid peptides

Some of the most potent neuropeptides are opioids that target respective opioid receptors (OPs), which include the μ (MOP), δ (DOP), κ (KOP), and nociceptin/orphanin FQ (NOP) classes (Henry et al., 2017). These GPCRs are expressed amongst a wide distribution throughout the central and peripheral nervous systems and multiple organ systems. The enkephalins play a role in analgesia, the stress response, peristalsis, the cardiovascular system, and neuroprotection, amongst many other functions (Hughes et al., 1975). In the CNS, these receptors are responsible for pain mediation via neuronal neurotransmitter release and postsynaptic hyperpolarization and are also expressed by both microglia and astrocytes (Eriksson et al., 1993; Liang et al., 2014; Gavioli et al., 2015; Corkrum et al., 2019; Maduna et al., 2019; Machelska and Celik, 2020; Reiss et al., 2021; Xu et al., 2023).

#### 2.2.1 Endogenous opioid enkephalins

Endogenous opioid enkephalins (ENK) are peptide derivatives of the endorphin family that signal through various OPs, including the MOP peptide receptor and the DOP opioid peptide receptor (Gupta et al., 2021). Enkephalins are pentapeptides derived from the precursor proenkephalin (PENK) and are subdivided into two groups based on the amino acids present on their carboxy-terminal, either leucine or methionine (Cullen and Cascella, 2024). These are the leu-enkephalins (LENK) and the met-enkephalins (MENK) (Reiner, 1987). Qin et al. demonstrated that pre-treatment of neuron-glia cultures with LENK had a neuroprotective effect on dopaminergic neurons against LPS-induced neurotoxicity via attenuation of the reactive oxygen species (ROS) mediated amplification of TNFα in microglia as well as the inhibition of microglial NADPH oxidase mediated damage (Qin et al., 2005). LENK also boosted both cell viability and induction of autophagy in astrocytes exposed to oxygen-glucose deprivation, exhibiting a cryoprotective effect in the central nervous system (Wang et al., 2019). Stiene-Martin and Hauser (1990) demonstrated a total decrease in cell numbers of astrocytes of mixed glial cultures treated with MENK as compared to basal cell treatments and those treated with naloxone, an opioid-receptor antagonist, indicating MENK-mediated suppression of astrocyte growth in culture. MENK treatment not only induced microglia to the M1 phenotype via significantly increased levels of M1-associated genes including TNFα, IL12, and cluster differentiation 40 and 86 (CD40 and CD86) but also boosted microglial cytotoxicity toward glioblastoma cells, indicating the peptide's functional relevance regarding the polarization of microglial phenotypes as well as its tumoricidal properties (Xu et al., 2016).

Although LENK can have a degree of specificity to DOP, these peptides are small and may suffer from poor bioavailability (Mosberg et al., 2014). The desire to generate longer-lasting PENK peptides with unique specificities led to the generation of DADLE, capable of binding to DOP and the one subset of MOP, as well as DAMGO and DPDPE, which are specific to MOP and DOP, respectively. Like other DOP agonists, DADLE and DPDPE produce an anti-inflammatory response in microglia and generate a cyto/neuroprotective response from astrocytes (Liang et al., 2014; Shrivastava et al., 2017; Wang et al., 2019; Mali and Novotny, 2022). DAMGO, like other MOPs, stimulates a pro-inflammatory outcome with higher rates of glutamate-mediated gliotransmission (Corkrum et al., 2019; Cuitavi et al., 2023). The amphibian-derived-PENK dermorphin and its derivative DALDA are also potent targets for the MOPs. However, they have demonstrated potent inhibition of neuroinflammatory cascades and oxide-nitrosative stress through down-regulation of Trp ion channels in a chemotherapy-induced neuropathic pain rodent model (Gadepalli et al., 2023). Other noteworthy PENKs have been discovered from amphibians, including dermenkephalin or deltorphins, which include deltorphin A, I, and II. As their name suggests, their excellent affinity to DOPs has had noteworthy effects on the glial mitigating of neuropathic cold allodynia (Mika et al., 2014; Reiss et al., 2021). Astrocytes were the most affected by the deltorphins as Mika et al. showed that DOP expression in primary microglia was lacking, suggesting that DOP neuroinflammatory pain mediation might require the cooperative effort of mixed glial interactions. These peptides have rapidly become stand-ins for OP specificity studies. How these peptides interact with OPs may necessitate further study, as the expected mediation of inflammation is not always clear.

#### 2.2.2 Prodynorphins

The prodynorphins (PDYN) are also a class of PENK-proenkaphalin B opioid polypeptides. Of these, endomorphin 1 and 2 are tetrapeptides capable of having an inhibitory function on MOPs, which leads to the regulation of neuroinflammatory functions in microglia, including phagocytosis, chemotaxis, and the secretion of superoxide anions (Azuma et al., 2001; Lu et al., 2022). Dynorphins A and B are PDYNs uniquely specific to KOPs and capable of mediating neuroinflammation and pain through microglia and astrocytes (Liu et al., 2020; Lee et al., 2022). PDYNs also include endorphins, of which the MOP specific β-Endorphin inhibits microglial neuroinflammation comparable to IL10 (Mao et al., 2019; Ma et al., 2021; Belo et al., 2023), which is considered one of the more potent inflammatory regulating cytokines (Carlini et al., 2023). The dynorphin peptide α-neoendorphin can bind to MOP, DOP, and KOP but has only been observed to regulate pain when secreted by spinal astrocytes (Wahlert et al., 2013).

#### 2.2.3 Prepronociceptins

The nociceptin receptors (NOPs) are involved in emotional and pain regulation and have also been discovered in microglia and astrocytes (Brown and Cox, 2006; Meyer et al., 2017). The opioids specific to MOP, DOP, and KOP are usually not effective on NOPs. The primary agonists of NOPs are prepronociceptin (PNOC) peptides, with Nociceptin/ Orphanin (N/OFQ) being the natural peptide used in neuropeptide signaling. In astrocytes, N/OFQ regulates glutamate/aspartate homeostasis in maturation and development (Meyer et al., 2017). N/OFQ has also been shown to attenuate interleukin one beta (IL1β) in astrocytes and microglia due to either LPS agonism of TLR4 or traumatic injury (Mallimo and Kusnecov, 2013). The NOP does see an increase in LPS and PD models of microglia (Brown and Cox, 2006), suggesting there may be a natural inflammatory mediation due to this pathway. Although these receptors are prevalent in microglia, which are known to be involved in pain regulation, the role of NOPs in microglial pain mediation is still unclear (Machelska and Celik, 2020).

### 2.3 Bombesins

The bombesins (BB) are a class of neuropeptides that target the GPCRs called bombesin receptors (BBR1, 2, and 3), which owe their name to the first bombesin peptide discovered from the skin of the European fire-bellied toad *Bombinabombina* (LaPelusa and Jan, 2024). BB is known chiefly for its exogenous effects on appetite suppression in the CNS. However, gastrin release peptide (GRP) and neuromedins (NMB, NMC, and NMU) were later discovered to be the mammalian peptide of the bombesin family (Majumdar and Weber, 2012). GRP has a role in pain modulation and signaling for astrocytes related to chronic itch (Ji et al., 2019), which microglia can exacerbate (Liu et al., 2023). NMB has a role in astrocyte-mediated depolarization of CA1 pyramidal neurons by increasing the number/amplitude of small inhibitory postsynaptic currents (Lee et al., 1999). Mason et al. (2002) compared NMB to NMC, which was able to cause astrocytes to hyperpolarize (higher amplitude and frequency) with Ca^2+^ 32-fold higher in NMC. NMU, although abundantly secreted in the hippocampus, functionality might be linked to protection against neuroinflammation. Iwai et al. (2008) demonstrated the promotion of neuroprotection against mixed glial (astrocytes and microglia) LPS conditioning with increased BDNF secretions and sparing rodent memory loss.

### 2.4 Pancreatic peptides

Neuropeptide Y (NPY) belongs to the family of pancreatic peptides, which includes both peptide YY and pancreatic polypeptide (PP) (Holzer et al., 2012). PP and Peptide YY have been studied concerning the gut-brain axis and the regulation of appetite and obesity (Karra et al., 2009; Holzer et al., 2012). NPY has a wide distribution in both the PNS and CNS and has diverse functional involvement in feeding behaviors, blood pressure control, memory, anxiety, and circadian rhythms (Thorsell and Heilig, 2002). Ferreira et al. (2010) demonstrated NPY-mediated inhibition of NO, IL1β, and subsequent NFκB activation in N9 murine microglia exposed to LPS. NPY also exhibited protective effects toward N9 microglia and a reduction of CD11b immunoreactivity in hippocampal microglial cultures exposed to methamphetamines, suggesting a neuroprotective role of NPY in meth-induced microglial injury (Gonçalves et al., 2012). Li et al. (2014) found that in rat microglia exposed to LPS and co-treated with NPY, there was a reduction in LPS-induced increases in TNFα, IL1β, and N-methyl-D-aspartate (NMDA) current excitotoxicity via inhibition of microglial reactivity. NPY is also expressed in astrocytes, and Barnea et al. demonstrated an increase in both proNPY-mRNA and production of NPY in cultures exposed to IL1β, suggesting immune involvement of this peptide in astrocytic inflammatory pathways (Barnea et al., 2001).

Amylin is a peptide hormone that has co-secretion with insulin from the beta-cells of the pancreas (Ludvik et al., 1997). Functionally, its primary role is a glucoregulatory hormone, with significant importance in energy/glucose metabolism, inhibition of glucagon secretion, satiety, and delayed gastric emptying (Hay et al., 2015). Amylin easily traverses the blood-brain barrier (BBB) and plays a part in a variety of functions in the CNS, including a role in appetite inhibition, the relaxation of cerebrovascular structures, and its potential for neural regenerative capabilities (Westfall and Curfman-Falvey, 1995; Trevaskis et al., 2010; Roth, 2013). In a transgenic model of AD, administration of an amylin antagonist reduced inflammatory microglial markers, including ionized calcium binding adaptor molecule 1 (Iba1) and CD68, caspase-1, TNFα, and IL1β, and a concordant reduction in Aβ plaque burden and size compared to controls, implicating amylin in the pathogenesis of AD (Fu et al., 2017). Wang et al. demonstrated that peripheral amylin treatment reduced levels of the inflammatory markers CD68 and Iba1—two markers directly correlated with the levels of neurofibrillary tangles in AD's models—via amylin's effect on CD68 in microglial cells (Wang E. et al., 2017).

Additionally, administration of an amylin antagonist improved learning and spatial memory deficits in AD mice, with associated suppression in microglial activation and neuroinflammation (Soudy et al., 2017). Amylin was also shown to produce effects in astrocytes that mimic those of Aβ proteins, such as the inhibition of astrocytic redox activity and the induction of reactive gliosis (Abe et al., 1997). Collectively, these studies indicate amylin and its resultant receptor cascades as another promising therapeutic target for future AD treatments.

### 2.5 Intestinal peptides

Vasoactive intestinal peptide (VIP) is a 28 amino acid peptide from the secretin and glucagon family. Initially, it was found to have vasodilatory effects, and it has a wide distribution in the CNS and peripheral nervous system (PNS), cardiopulmonary, reproductive, and digestive systems (Iwasaki et al., 2019). VIP also shares 68% sequence similarity with neuropeptide pituitary adenylate cyclase-activating polypeptide, also known as PACAP (Moody et al., 2011). PACAP is a 38-amino acid peptide mainly found in the hypothalamus and has been used as a biomarker for TBI (Toth et al., 2020). These peptides act as ligands for many of the same GPCRs, including PAC1, VPAC1, and VPAC2 (Carniglia et al., 2017). Both PAC1 and VPAC1 have been detected in rat microglia, and VIP and PACAP reduced TNFα mRNA production in cultured microglia exposed to LPS in a cyclic adenosine monophosphate (cAMP) dependent fashion (Kim et al., 2000). Delgado et al. have examined these peptides in a variety of studies. They found that VIP and PACAP reduced pro-inflammatory cytokine production of nitric oxide (NO), TNFα, IL1β, and IL6 in LPS-exposed microglia (Delgado et al., 2003). In a PD study with mice exposed to neurotoxic 1-methyl-4-phenyl-1,2,3,6-tetrahydropyridine (MPTP), VIP treatment produced a significant reduction in the loss of dopaminergic neurons in the substantia nigra. It reduced MPTP-induced microglial activation and release of inflammatory cytokines. VIP also reduced Aβ plaque-induced neurodegeneration in activated microglia by p38 mitogen-activated protein kinase (MAPK), p42/p44 MAPK, and NFkB signal blockade (Delgado et al., 2008).

PACAP-27, a 27-amino acid-based derivative, reduced neurodegenerative in CA3 pyramidal neurons via reduced microglial activation and boosted learning/memory performance in aged mice treated with neurotoxic prostaglandin (Avila et al., 2019). PACAP-38 was found to modulate the inflammatory response by reprogramming microglia into the M2 phenotype in a degenerative retinal animal model (Denes et al., 2023). Additionally, PACAP-38 reduced neuronal damage via attenuation of IL6 and reduction of mitochondrial cytochrome c release in an ischemic mouse model (Ohtaki et al., 2006). VIP and PACAP induced increased activity-dependent neuroprotective protein (ADNP) in cortical astrocytes from newborn rats in a VPAC2-dependent mechanism (Zusev and Gozes, 2004). In astrocytes exposed to PACAP, the maximal velocity of glutamate uptake was increased, with a similar effect also produced on exposure to higher concentrations of VIP, suggesting these two peptides are implicated in glutamate-mediated neuropsychological disorders. A comprehensive review of the role of both PACAP and VIP regarding the functions of astrocytes can be found in the work of Masmoudi-Kouki et al. with implications evidence in glial cell activity and proliferation, glycogen metabolism, cell plasticity and release of neuroprotective factors (Masmoudi-Kouki et al., 2007).

Glucagon-like peptide 2 (GLP-2) is another peptide derivative of the proglucagon gene. It is a member of the glucagon-like peptide family of intestinal hormones known for their diverse roles in intestinal function and growth, gastric motility, and acid secretion, and regulation of appetite and energy homeostasis, amongst other functions (Burrin et al., 2001). These actions are mediated via the GLP-2 receptor (GLP-2R) in a GPCR-mediated cascade (Drucker, 2001, p. 2). The GLP-2 receptor has been localized within several regions of the mouse and rat CNS, including but not limited to the cerebellum, amygdala, cerebral cortex, and hippocampus (Lovshin et al., 2001). Velázquez et al. (2009) demonstrated a synergistic effect of GLP-2 on the proliferation of rat astrocytes, DNA synthesis, and astrocyte density, suggesting a regenerative role for GLP-2 in glial cells of the CNS. In a PD mouse model, a GLP-2 analog reduced the inflammatory response by modulating microglial activation and reducing levels of inflammatory cytokines. It improved the mitochondrial dysfunction induced by MPTP within the substantia nigra (Zhang et al., 2021).

Exendin-4 (Ex-4) is a GLP-1R agonist that is found within the saliva of the Gila monster lizard. GLP-1 affects insulin secretion, the inhibition of food intake, gastric emptying, and glucagon secretion (Ding et al., 2006). The effect of GLP-1 is limited due to rapid breakdown by peptidases, limiting its clinical applications. Ex-4 is a more stable GLP-1 analog used for treating type II diabetes and has also been found to cross the blood-brain barrier and exhibit neuroprotective effects within the CNS. In an MPTP mouse model of PD, treatment of Ex-4 systemically prevented microglial activation induced by MPTP and release of pro-inflammatory mediators such as TNFα and IL-1β, suggesting Ex-4's role as a potential therapeutic in neurodegenerative diseases (Kim et al., 2009). In astrocyte cultures exposed to oxygen-glucose deprivation, treatment with Ex-4 reduced levels of inflammatory mediators derived from astrocytes in ischemic brain tissue after middle cerebral artery occlusion and mitigated the resultant breakdown of the BBB (Shan et al., 2019).

Uroguanylin—a member of the natriuretic family of guanylin peptides—is an intestinal peptide hormone most prominently found in the epithelium of the gastrointestinal tract with a diverse impact on physiological processes, including its role in digestive fluid secretions and renal salt balance (Forte, 2004a). Uroguanylin activates guanylate cyclase C (GC-C), particularly in the intestine, where GC-C is an enterotoxin receptor. GC-C has also been characterized in other non-intestinal tissues such as the kidneys, lungs, reproductive system, and the brain (Forte, 2004b). In the CNS, GC-C is localized within the dopaminergic neurons of the midbrain and ventral tegmental area. GC-C activation heightens glutamate and acetylcholine receptor-mediated excitatory responses, and GC-C knockout mice develop attention deficits and hyperactive behavior (Gong et al., 2011). Uroguanylin also has a GC-C-independent mechanism via modulation of Ca^2+^ in astrocytes by influencing their intracellular pH (Habek et al., 2021). This suggests that uroguanylin plays a role in Ca^2+^-mediated signaling pathways in astrocytes, such as regulating neuronal circuits (Guerra-Gomes et al., 2017).

### 2.6 Statins

Somatostatin (SST) is a neuropeptide of the statin family that exists in a cyclic form due to its disulfide bonds between cysteine residues and acts as an endocrine and exocrine inhibitor in systems, including the liver, lungs, pancreas, gastrointestinal tract, adrenals, and thyroid (O'Toole and Sharma, 2024). SST has two molecular formulations known as somatostatin 14 (SST-14) and somatostatin 28 (SST-28), based on the presence of 14 or 28 amino acids that are derivatives from proteolysis from a precursor molecule, pre-pro-SST (Barbieri et al., 2013). These molecules interact with five subtypes of GPCRs known as sst1-sst5. Not only is the presence of mRNA for several of these receptors—sst2, sst3, and sst4— characterized in primary cultured rat microglia, but SST is functionally active and inhibits microglial activation via reduced IL-3 and granulocyte-macrophage colony-stimulating factor (GM-CSF) signaling (Feindt et al., 1998). Bai et al. examined the effects of SST in a PD in which LPS was injected into the substantia nigra of rats. SST treatment reduced the LPS-induced loss of dopaminergic neurons in the substantia nigra due to suppressed activation of microglia and the NFkB pathway. Additionally, there was decreased production of TNFα, IL1β, ROS, and prostaglandin E2, known to be produced by active microglia in PD (Bai et al., 2015). SST was also found to boost the production of insulin-degrading-enzyme (IDE) in rat microglia and mouse BV2 cells. IDE is the main extracellular protease secreted by microglial cells to degrade Aβ plaques, suggesting that SST levels in microglia may be crucial to the pathogenesis of AD (Tundo et al., 2012). SST is commonly co-expressed with Aβ, although SST does not typically co-express with astrocytes despite sharing a similar spatial distribution in the CNS (Gonzalez-Rodriguez et al., 2021). In 2020, Hernandez et al. examined the effect of administering topical SST in a mouse model of diabetic retinal neurodegeneration. They found SST exhibited an anti-inflammatory effect by inhibiting the inflammatory M1 microglial response to an LPS trigger (Hernández et al., 2020). Likewise, Mazzeo et al. demonstrated a reduction of pro-inflammatory and pro-apoptotic mediators in human retinal pericytes exposed to microglial cells treated with LPS and SST vs. LPS alone (Mazzeo et al., 2017).

Despite its vast array of functions, somatostatin's clinical efficacy was reduced by shortcomings related to its short half-life, necessity for intravenous administration, and hypersecretion of hormones such as insulin, glucagon, and growth hormone after SST administration (Yuen and Samson, 2022). Due to this, SST analogs such as octreotide were developed. Octreotide is a cyclic SST analog with different receptor-binding characteristics. It has an improved half-life of 2-h compared to SST's half-life of 3 min and does not cause rebound hypersecretions of hormones. Feindt et al. demonstrated that octreotide inhibits microglial activation in their study alongside SST via reduced IL3 and GM-CSF signaling (Feindt et al., 1998).

Cortistatin is another cyclic neuropeptide belonging to the somatostatin family expressed in 14 and 17 amino-acid isomers known as cortistatin 14 (CST-14) and cortistatin 17 (CST-17). These two peptides share 11 amino acids with SST-14 and can bind the same SST receptor motifs with an affinity similar to that of SST (de Lecea, 2008). Despite shared homology with SST receptors, cortistatin has unique effects in the CNS that are different from SST, including its role in locomotor activity and slow-wave sleep induction, suggesting alternative signaling pathways distinct to CST (Carniglia et al., 2017). In addition, evidence suggests that CST plays a role in neuroinflammatory pathways. In the CNS, cortistatin-deficient mice were predisposed to weakening of the endothelium, disruption of tight junctions, BBB leakage, and dysregulation of immune activity that was reversible upon treatment with cortistatin in an *in-vitro* barrier model simulating an ischemic environment (Castillo-González et al., 2023). In a well-established model of PD based on exposure to the neurotoxic compound MPTP, cortistatin treatment improved locomotor activity and reduced glial activation in affected brain regions, reduced the production of inflammatory mediators, and boosted the production of neurotrophic factors in the striatum (Serrano-Martínez et al., 2024). These anti-inflammatory and neuroprotective properties of cortistatin indicate the peptide's potential as a novel therapeutic agent in treating PD.

### 2.7 Kinins

Tachykinins are a group of peptide hormones that have diverse expression throughout both the nervous and immune systems. The main mammalian tachykinins include substance P (SP), neurokinin A (NKA), neurokinin B (NKB), neuropeptide K (NPK), and hemokinin-1 (HK-1). The tachykinins signal utilizes three neurokinin receptor subtypes: NK-1R, NK-2R, and NK3-R (Steinhoff et al., 2014). Of the tachykinins, SP has been widely examined with many different pathophysiological effects, including roles in nociception, regulation of bone metabolism, and inflammatory bowel disease. Human microglial and astrocytic cells both express robust amounts of the NK-1R isoform, which is functionally augmented by SP in inflammatory and neurotoxic glial responses (Burmeister et al., 2017). SP has been shown to induce pro-inflammatory cytokines and stimulate immune cells in an NFkB-mediated manner (Johnson et al., 2017). In neurogenic inflammation, the SP receptor NK-1R has been found in human fetal microglia (Lai et al., 2000). Martin et al. (1993) examined that SP alone did not enhance IL1 or TNFα production in rat microglia but found that SP and LPS synergistically quadrupled the release of IL1β compared to LPS by itself, suggesting an implication for SP in inflammatory pathology within the CNS. More recently, Zhu et al. demonstrated that SP can induce the activation of microglia and the subsequent release of pro-inflammatory factors such as Il1 and TNFα in primary cultured microglia (Zhu et al., 2014). SP also has a role in microglial density in chemotaxis of the substantia nigra (Wang Q. et al., 2015). Additionally, SP potentiated increased class II major histocompatibility protein expression in the microglia of rats treated with interferon-gamma (McCluskey and Lampson, 2001). SP is found at a high concentration within the substantia nigra, suggesting that this peptide might be implicated in the pathology of PD, especially considering that SP potentiates the release of dopamine in the striatum. Block et al. examined that SP-activated nicotinamide adenine dinucleotide phosphate (NADPH) oxidase in microglial cells, subsequently producing excess intracellular ROS and extracellular superoxide that was neurotoxic to dopaminergic neurons in a microglia-dependent manner (Block et al., 2006). Inhibition of the NK-1R receptor attenuated both the microglial inflammatory response process and dopaminergic neurotoxicity induced by LPS-activated BV2 microglia, further suggesting the role of SP in PD (Jiang et al., 2020).

Hemokinin-1 (HK-1) is another member of the tachykinin family, named for its unique role in the hematopoiesis of B-lymphocytes (Zhang et al., 2000). Encoded by the Tac4 gene, HK-1 is the only tachykinin peptide primarily produced outside neural tissues. Sakai et al. discovered that the Tac4 tachykinin gene is predominantly expressed in primary cultures of microglia in comparison to the Tac1 gene that encodes SP. Tac4 mRNA expression in the microglial cells was also upregulated in response to LPS compared to the Tac1 gene, suggesting HK-1 may play a more prominent role in microglial activation in the CNS with SP in neuroinflammatory disorders relating to pathological microglial activation (Sakai et al., 2012). In a model of inflammatory orofacial pain within the trigeminal ganglia, Tac4 was upregulated in neurons and satellite glial cells, suggesting a role for HK-1 release in neuroglial interactions under inflammatory conditions within the CNS (Aczél et al., 2020).

Cholecystokinin (CCK) is a 33-amino acid long peptide hormone found abundantly within the gastrointestinal tract and the CNS. CCK is implicated in regulating nociception, feeding, and learning/memory by interacting with two G-protein coupled receptors known as CCK1 and CCK2 (Okonkwo et al., 2024). CCK has a variety of isoforms, including cholecystokinin octapeptide (CCK-8), which is abundantly expressed within the CNS (Rehfeld, 2021). CCK has been studied in the context of neurodegenerative diseases such as AD, with a relationship found between higher levels of CCK and reduced probability of mild cognitive impairment and AD (Plagman et al., 2019). Intestinal protein level expression of CCK was reduced significantly in a PD model, indicating a possible role of CCK in the gut-brain axis of PD pathology (Choi et al., 2021). In an aged mice model of delayed neurocognitive recovery, CCK-8 treatment reduced the activation of microglia and A1 reactive astrocytes and suppressed the expression of inflammatory mediators in the hippocampus (Chen et al., 2021). Müller et al. (1997) demonstrated that CCK-8 induced repetitively increased calcium signaling in rat and mouse hippocampal astrocytes, signifying astrocytes as a significant target for CCK in the CNS. CCK-8 also suppressed methamphetamine-induced microglial activation and production of both IL6 and TNFα both in vitro and *in vivo* (Gou et al., 2020).

Another peptide involved in the kinin system is known as bradykinin, which is a vasoactive peptide that is of particular importance in relation to inflammatory reactions and the regulation of blood pressure (Pirahanchi and Sharma, 2024). Functionally, bradykinin can increase vascular permeability and vasodilation of the gastrointestinal system, along with the urethra, uterus, and aorta. Bradykinin is also implicated in various pathophysiological conditions such as hereditary acquired angioedema, hypertension, allergic respiratory reactions, and AD, among others (Golias et al., 2007). These biological effects are mediated by specific GPCRs known as B1 and B2. In the CNS, bradykinin is released endogenously after injury or stroke. It can interact with bradykinin receptors on microglia to induce an anti-inflammatory cascade and subsequently produce a neuroprotective effect. Bradykinin has also been demonstrated to attract microglia to the site of a CNS lesion and boost microglial motility *in vitro* within mixed cultures of cerebrocortical cells of rats. This effect was reversed with a B1 receptor antagonist (Huisman et al., 2008). Liu et al. demonstrated that the bradykinin-mediated stimulus of B2 receptors induced increased Ca^2+^ signaling and generation of ROS in astrocytes. The increased ROS boosted the downstream release of glutamate from these astrocytes, which subsequently interacted with NMDA receptors and increased Ca^2+^ in the cytosol of neurons, indicating bradykinin's role in the crosstalk between astrocytes and neurons within the CNS (Liu et al., 2009). In BV2 microglial cells, a B1 receptor antagonist caused a significant increase in LPS-induced nitric oxide release nitric oxide synthase production, and TNFα release. Additionally, intranasal administration of the B1 receptor antagonist increased the amyloid plaque burden and accumulation of microglia in the cortex of mice in an AD model, indicating that bradykinin has a role in neuroinflammatory diseases (Asraf et al., 2017).

### 2.8 Galaninergic peptides

Galanin (GALA-2-11) is a 30-amino acid-long regulatory peptide with a diverse distribution within the central and peripheral nervous system that functions in a variety of physiological states, including feeding and gastrointestinal motility, nociception, learning/memory, and epileptic activities within the brain (Vrontakis, 2002). These functions are mediated by interactions with three GPCRs known as GAL_1 − 3_-R. GALA-2-11 was demonstrated to boost the induction of c-Fos, JunB, and Tis11 mRNA production within cultured astrocytes, indicating that there are functional GALA-2-11 receptors on neuroglial cells in the CNS (Priller et al., 1998). Koller et al. (2019) demonstrated that exposure to exogenous GALA-2-11 influenced the differentiation/polarization of macrophages by modulating the expression of chemokines and inflammatory cytokines such as TGF-β, IL-10, and IL-1Ra, most prominently in Type-1 macrophages. Additionally, the reduced mRNA of the Gal-1-receptor (GALR1) via null mutation or administration of galantine—a GALR1 antagonist—in a mouse model of status epilepticus was found to increase damage within the hippocampus, suggesting a role for the GAL1R as a potential therapeutic target in modulating cell death during epileptic insults within the CNS (Schauwecker, 2010).

### 2.9 Adrenal peptides

Adrenomedullin (AM) and calcitonin gene-related peptide (CGRP) are neuropeptides of the CGRP/calcitonin family. AM was initially isolated from the adrenal gland, has a significant vasodilatory effect, and plays vital regulatory roles in the cardiovascular, renal, and lymphatic systems (Schönauer et al., 2017). Over-expression of AM promotes astrocyte mediated neuroprotection in a cerebral ischemic stroke (Xia et al., 2004). CGRP and AM were demonstrated to reduce LPS-induced microglial activation *in vitro* and reduce pro-inflammatory mediators such as IL6, TNFα, and NO (Consonni et al., 2011). In a model of experimental autoimmune encephalomyelitis (EAE), ADM reduced the clinical severity of EAE, decreased the production of inflammatory mediators in microglia and astrocytes such as IL6, IL12, and TNFα, and boosted the expression of neuroprotective factors such as BDNF and activity-dependent neuroprotector protein (ADNP) (Pedreño et al., 2014). In a different model of chronic murine EAE, CGRP also reduced the clinical severity of disease and attenuated microglial activation (Sardi et al., 2014). Together, this evidence suggests the role of both AM and CGRP in the modulation of microglial activation in neuroinflammatory diseases. CGRP induces the production of both microglia and astrocytes at the transcription level, and increases the release of tissue plasminogen activator, implicated in the tissue remodeling process. This suggests a role for CGRP in glial activation during motor neuron injury (Reddington et al., 1995). Intrathecal administration of CGRP was also demonstrated to increase the number of astrocytes that act on receptors within these astrocytes and lead to H3K9 acetylation—associated with inflammatory gene expression, proliferation, and autophagy. Increased amounts of astrocytes with higher amounts of H3K9 acetylation are seen after nerve injuries, suggesting a role for CGRP in attenuating neuropathic pain in an astrocyte-mediated mechanism (Sun C. et al., 2021).

Intermedin—also known as adrenomedullin-2 (AM2)—is a peptide related to CGRP and adrenomedullin, with shared overlap and homology in various receptor activities (Roh et al., 2004). AM2 is primarily expressed in the gastrointestinal system, the pituitary gland, and the renal system. AM2 plays a significant role in water and electrolyte balance, systemic and pulmonary vasodilation, and cardiac contractility (Bell and McDermott, 2008; Cui et al., 2008). AM2 inhibited inflammation induced by LPS in BV2 microglial cells via the reduction of inflammatory mediators, including TNFα, IL1β, cyclooxygenase 2 (COX2), and inducible nitric oxide synthase (iNOS) (Sun et al., 2021). AM2 also exhibited an antioxidant effect within the hippocampus and modulated inflammation in BV2 microglial cells. It inhibited the nuclear translocation of inflammatory mediators, including NFκB p-65 and nuclear factor of kappa light polypeptide gene enhancer in B-cells inhibitor alpha (IκBα). Additionally, AM2 reduced the generation of ROS, indicating its neuroprotective properties (Lee et al., 2023). This collectively suggests the role of AM2 in neuroinflammatory diseases and indicates this peptide's potential as a future therapeutic target.

### 2.10 Adipose regulators

Leptin (LEP) is a peptide hormone mainly known for its involvement in energy and appetite homeostasis. It is produced predominantly in adipose tissues, with LEP expression varying with changes in nutritional states (Ramos-Lobo and Donato, 2017). LEP is too large of a molecule to cross the BBB and is transported centrally via a regulated transport system. However, some studies suggest that LEP can be produced in the CNS in areas such as the hypothalamus, cortex, and cerebellum, indicating there may be local, specific functions of this hormone (Morash et al., 1999). Both the short (LEPRa) and long (LEPRb) isoforms of the LEP receptor were detected in primary cultures of mouse microglia, and LEP treatment resulted in a dose-dependent increase in IL1β via a STAT3-dependent mechanism (Pinteaux et al., 2007). Leptin knockouts in mouse astrocytes resulted in mediated hypothalamic pSTAT3-related hypothatlamic gliosis and excacerbated diet-induce obesity (Wang Y. et al., 2015). Previously, pharmacologic blockage of microglial phagocytosis in obese mice reduced obesity-associated cognitive decline and dendritic spine loss (Cope et al., 2018). Ma et al. demonstrated that LEP treatment reduced Aβ plaque burden, increased microglial immunoreactivity, and increased both IL1β and IL6 levels in the hippocampus of adult mice compared to the saline control group, implicating LEP signaling in microglial activation and the release of central inflammatory mediators (Ma et al., 2023). In a rat model of spinal cord injury (SCI), LEP administration reduced microglial activation and boosted functional recovery (Fernández-Martos et al., 2012). In a mouse model of adult-onset obesity, obesity receptor (OBR)—the primary LEP transport receptor at the BBB—exhibited significantly increased immunofluorescent staining within astrocytes of the hypothalamus of the obese mice as compared to the control, suggesting astrocyte's role in the pathogenesis of obesity within the CNS (Pan et al., 2008).

Obestatin is a 23-amino acid long gastrointestinal peptide hormone with a diverse range of physiological functions, mainly known for its role in the reduction of both food intake and weight gain (Cowan et al., 2016). It operates along the gut-brain axis and is also involved in regulating sleep, memory improvement, secretion of pancreatic enzymes, and blood pressure regulation (Lacquaniti et al., 2011). Obestatin also exhibits a cardioprotective effect via inhibition of apoptosis in reperfusion injuries within models of cardiac ischemia (Alloatti et al., 2010). In the CNS, obestatin exerted a neuroprotective effect and antioxidant properties against ischemic injury via attenuation of astrocyte activation and reduction of neuronal cell apoptosis (Mirarab et al., 2019).

### 2.11 Growth hormone secratogues

Growth hormone secretogues (GHS) are neuropeptides that induce the secretion of growth hormones. They were discovered in studies attempting to understand the underlying causes of endocrine-related aging and are crucial in maintaining metabolic radicals (Poudel et al., 2020; Cai, 2021; Tresguerres et al., 2022). Ghrelin or acylated Acyl-Ghrelin (AG) is a GHS that is involved in hunger regulation but also has an -inflammatory effect on microglia involved in NLRP3 inflammasomes, pyroptosis, and the NFκB and TNFα pathways (Liu et al., 2019; Maldonado-Ruiz et al., 2019). In astrocytes, AG is involved in glucose and glutamate homeostasis and metabolism (Fuente-Martín et al., 2016; Frago and Chowen, 2017). The endocrine-secreted growth hormone-releasing factor's (GRF) primary function is to bind to receptors and induce the production of growth hormone; however, this peptide is produced across many other systems, including the reproductive and immune systems. In the ocular system, GRF has been noted as an inflammatory mediator lending to the protection of ocular neurons (Cen et al., 2022). Other GHSs exist, such as insulin-like growth factor; however, there is limited understanding of their interaction with neuroglia, to date.

### 2.12 Other hormone peptides

Some neuropeptides significantly affect glial and overall endocrine signaling related to social bonding and stress. Oxytocin (OXT) is a hormonal peptide that affects maternal bonding and reproduction (Lee et al., 2009). Although the OXT receptor is not entirely understood in glia, OXT does have a role in neuroinflammation and glial signaling. When treated with microglia, it inhibits inflammation *in vitro* and *in vivo* with AD Aβ plaque models (Selles et al., 2023). With astrocytes, OXT is crucial in regulating neuromodulation, gliotransmission, and neural plasticity (Wang P. et al., 2017; Baudon et al., 2022). Vasopressin/arginine vasopressin (VPN/AVP) is a neuropeptide with a significant role in social bonding and human reproduction and has a complementary function to OXT. Like OXT, VPN has a role in neuroinflammation, being secreted by reactive microglia (Szmydynger-Chodobska et al., 2011) or exacerbating microglial into pro-inflammatory cytokine production in a TBI rodent model (Szmydynger-Chodobska et al., 2010). Fundamentally, VPN is responsible for osmotic regulation in a variety of cells. In astrocytes, this is characterized by swelling and uptake, which remains functionally unclear (Sarfaraz and Fraser, 1999). Apelin (APJ) is a peptide that targets the apelin GPCR, is related to body fluid uptake and appetite, and reduces the secretion of VPN. In rats with SCI, an APJ's attenuation on microglial and astrocyte-mediated neuroinflammation promoted the survival of endogenous neural stem cells (Liu et al., 2022). Apelin-13 (APEL) was able to limit a neuroinflammatory response in lower NF-κB and IκB kinase β (IKKβ) levels in depressive rodents (Zhang et al., 2019), LPS stimulated N9 microglia, and astrocytes, and microglia in LPS injected mice (Zhou et al., 2019).

## 3 Extracellular matrix glial peptides

Several peptides and peptide derivatives from ECM have been discovered to have some degree of effect on glia (Table 2). In neurons and glial cells, a membrane-linked glycoprotein known as the neural cell adhesion molecule (NCAM) is involved in cell-cell interactions and plays an important role in the development of the nervous system along with synaptic plasticity, learning, and memory (Weledji and Assob, 2014). NCAM modulates an intracellular signaling cascade with its interaction between the fibroblast growth factor receptor (FGFR) and downstream activation of kinases, including protein kinase C, phosphatidylinositol-3 kinase (PI-3), and extracellular signal-regulated kinase (ERK) (Niethammer et al., 2002). Fibroblast growth loop —or FGL—is an NCAM-derived peptide mimetic that has been shown to emulate the interaction between FGFR and influence cytokine levels in glial cells (Cox et al., 2013). Specifically, FGL promotes the production of IL4. This anti-inflammatory cytokine acts on neurons to induce the expression of a ligand at the neural cell membrane known as CD200, which provides a central inhibitory signal to the microglial response (Casali and Reed-Geaghan, 2021). In typical aging brains, there is a decline in IGF-1, a polypeptide hormone that inhibits the activation of microglia. This activation occurs through an IFNγ induced mechanism (Ivan et al., 2023). Downer et al. examined the interaction of FGL concerning imbalances among pro-inflammatory IFNγ and inhibitory IGF-1 levels in aging rat hippocampi with associated increased glial reactivity. FGL was shown to reverse the decline of IGF-1 in the aging neurons and promote CD200 and reduced antigen presentation of MHCII and CD40 expression in a robust manner, reducing IFNγ induced activation of glial cells that is more prominent during aging (Downer et al., 2009, 2010). Ojo et al. examined the effect of systemic FGL treatments on microglia and astrocyte population densities and activation in aging rat hippocampi (Ojo et al., 2011). In this study, FGL reduced the density of CD11b and MHCII-positive microglia and decreased glial fibrillary acidic protein (GFAP)-associated immunoreactivity in the subfields of all the aged hippocampi. Zellinger et al. (2014) showed new astrocyte growth and secretion of IL4 in their epileptic kindling electrode mouse model, which might be associated with promoting the development of a hyperexcitable network.

**Table 2:** Extra-cellular matrix-derived glial peptides.

**Type**	**Name**	**Sequence/Label**	**Study**	**References**
NCAM sequence	FGL	EVYVVAENQQGKSKA	Anti-inflammation in aged rat hippocampus	Ojo et al., 2011
			Anti-inflammation of MG in aged rats—IL4 and CD200 increase	Downer et al., 2009
			Anti-inflammation of MG in aged rats—IL4 increase and MHCII/CD40 decrease	Downer et al., 2010
			IL-4 secretion in demyelinating neurological diseases	Zellinger et al., 2014
	N/A	KHIFSDDSSE	Attenuation of AC proliferation	Sporns et al., 1995
			Selective AC adhesion on silica substrate	Kam et al., 2002
			AC mediation of MG adhesion on electrode surface	Sridar et al., 2017
	FRM	DRVEPYSSTA	Premyelinated OG viability	Palser et al., 2009
Laminin sequence	N/A	IKVAV	AC adhesion and reduced proliferation on silica substrate	Kam et al., 2002
			MG activation and brain biocompatibility	Koss et al., 2016
	N/A	YIGSR	AC adhesion on silica substrate	Kam et al., 2002
Fibronectin sequence	N/A	RGD	Neuroprotection via glia with NMDA treatment	Peluffo et al., 2007
			RG-like NSC isolation from fetal and adult mouse forebrain	Markó et al., 2011
Vitronectin	VDP	CGKKQRFRHRNRKG	AC differentiation from human iPSCs	Raman et al., 2020

Acronym nomenclature: AC, Astrocyte; CD40, cluster of differentiation 40; CD200, cluster of differentiation 200; FRM, fibroblast growth factor receptor activation motif peptide; FGL, fibroblast growth loop peptide; iPSCs, induced pluripotent stem cells; IL4, interleukin; MHCII, major histocompatibility complex II; MG, microglia; NCAM, neural cell adhesion molecule; NSC, neural stem cell; NMDA, N-methyl-D-aspartate; OG, oligodendrocyte; RG, radial glia; VDP, vitronectin-derived peptide.

NCAM and putative homophilic binding domain regions of NCAM (KHIFSDDSSE) have been shown to have an anti-proliferative effect on astrocyte cell division and growth (Sporns et al., 1995), which was later assessed on silica surfaces (Kam et al., 2002). Sridar et al. (2017) also measured astrogliosic attenuation when this peptide was coated on inert platinum-iridium electrode surfaces in a mixed glial 3D culture model. A fibroblast growth factor (FGF) receptor activation motif (FRM) peptide (DRVEPYSSTA) was modified into a multimeric form. It was shown to promote survival and outgrowth in myelinated oligodendrocytes (Palser et al., 2009). These studies demonstrate the novelty of NCAM peptide derivatives to promote myelin viability and attenuate the glial response in the context of reducing inflammatory neurodegenerative changes.

The basement membrane is an ECM that holds cells and tissues together, mainly due to the activity and function of laminins. These large glycoproteins (~400 kDa) are a central component of the basal lamina and have robust functions, including their involvement in the migration, adhesion, and attachment of cells (Aumailley, 2013). Laminins consist of three polypeptides linked by disulfide bonds that were initially known as A, B1, and B2 (Tashiro et al., 1989). The self-assembly of these proteins is essential in promoting basement membrane development, and their involvement in ECM affects the function of different neural cell types. For this reason, the sequences of their primary amino acids are being heavily studied. We will review the application and data on one such sequence known as IKVAV, a pentapeptide on the A chain of the laminin protein. It was shown that the adhesion of astrocytes on silica surfaces modified with IKVAV was not improved (Kam et al., 2002). The adhesion of astrocytes is critical in the early cell-to-substrate response, revealing that IKVAV plays a role in the attenuation of astrocytes and their subsequent functionality within the nervous system. In 2016, Koss et al. examined this sequence in the context of RADA_4_-IKVAV-derived nano scaffolds and their effect on primary microglia and astrocyte activity. It was found that IKVAV did not affect astrogliosis and microglial activation (Koss et al., 2016). YIGSR is another laminin-based peptide that was studied alongside IKVAV in the 2002 Kam et al. study, also shown to attenuate the adhesion of astrocytes on silica substrates. These results reveal that these laminin-based peptide motifs are involved in the bioactivity of microglia and astrocytes, especially concerning the adhesion of these cells in the early cell-substrate response.

Along with laminin, fibronectin is another large glycoprotein within the ECM. In contrast to laminin—which exists exclusively in the basement membranes—fibronectin is found in highly abundant organized structures within the membranes of both basement and interstitial ECMs. Fibronectin ranges in size from 230 to 270 kDa and is typically secreted by cells in a dimer form covalently linked by disulfide bonds (Dalton and Lemmon, 2021). It plays a significant role in cellular growth, differentiation, migration, and adhesion (Pankov and Yamada, 2002). Fibronectin self-assembles into a scaffold that initiates cell adhesion in a cell-mediated process in which this dimer is converted into a fibrillar network (Wierzbicka-Patynowski and Schwarzbauer, 2003). Due to its involvement in the ECM and cellular processes, amino acid sequences are being identified for their application in tissue engineering. One such sequence, RGD, has been studied regarding its application to neuronal nano scaffolds and adhesive biomaterials (Ruoslahti, 1996). Fibronectin binds to integrin receptors, which, aside from cellular adhesion, also mediate intracellular responses that support cellular survival and differentiation. This is a feature of embryonic radial glial cells, with large amounts of cellular surfaces interacting with fibronectin as the predominant ECM molecule (Campos, 2005). Markó et al. (2011) demonstrated that radial-glia-like neural progenitor cells had rapid adhesion to surfaces coated with an RGD motif peptide. In the CNS, molecules with RGD are induced following damage. In 2007, Peluffo et al. examined if these RGD motifs mediated neuroprotective effects after synthetic peptides containing RGD in a genetic vector were injected into rat brains within NMDA excitotoxicity models. They evaluated the effect of RGD on glial damage and inflammatory response. They found that the rats treated with the RGD peptide had significant increases in both microglia cell number and reactivity, although there was no difference in the response of astroglia (Peluffo et al., 2007).

Vitronectin is another glycoprotein in ECM-cell adhesion comparable to fibronectin but complexes with alternate specificity to laminin anchoring integrins (Schvartz et al., 1999). The RGD peptide is also present in vitronectin, yet other glial-responsive peptides are found in vitronectin (Raman et al., 2020). Specifically, Raman et al. used a vitronectin-derived peptide (VDP) to differentiate neural precursors into highly pure astrocyte populations effectively. The astrocytes were an effective model for inflammation with respect to Aβ uptake and apolipoprotein E (ApoE) production, which are hallmarks of AD.

## 4 Growth factor glial peptides

Both astrocytes and microglia are primary producers of growth factors for the development, maintenance, and repair of the CNS; however, a different panel of factors plays multiple critical roles in glial function. Transforming growth factor alpha (TGF-α) is crucial in maintaining glutamate transporter function in astrocytes, which provides neuroprotection associated with most neurodegenerative diseases (Karki et al., 2014). Several peptides associated with glial biology have been noted (Table 3). TGF-α is a 50 amino acid peptide with several cyclic loops (Tam et al., 1991), where the B loop beta sheet had the highest activity (Bettenworth et al., 2011). Other peptides based on the cyclic form of TGF-α were optimized with binding to monoclonal antibodies (Hahn et al., 2001). Transforming growth factor beta (TGF-β) variants 1, 2, and colony-stimulating factor 1 (CSF1) promotes proliferation and viability in microglia, and inhibitors have been designed with the intent of mediating inflammation associated with neuropathic and cancer pain (Bureta et al., 2019). The TGFβ1 binding domain mimicking peptide pm26TGF-β1 was used by Vaz et al. as a potent anti-inflammatory agent to favor IL10 over TNFα secretion and promoted differentiation in T cells while noting leukocyte rolling and neutrophil migration in their inflammatory mouse models (Vaz et al., 2015). The TGFβ1 inhibitor peptides P17 and P144 have been used with great effect to reduce SMAD phosphorylation and over-expression of collagens (Dotor et al., 2007; Hanafy et al., 2020), ameliorated inflammation and redox balance in a mouse ear injury (Murillo-Cuesta et al., 2015), and a fibrotic inhibitor across liver, skin scleroderma, and myocardial rodent models (Ezquerro et al., 2003; Santiago et al., 2005; Hermida et al., 2009). A capped CSF1 peptide was discovered alongside a tachykinin-like peptide in a systematic bioinformatic analysis. However, the *in vitro* potential of these peptides is yet unknown (Wiggenhorn et al., 2023).

**Table 3:** Glial-associated growth factor peptides.

**Type**	**Name**	**Sequence/label**	**Study**	**References**
CSF1	CAP-CSF1	pE-LLLPKSHSWGIVLPLGELE	Cell-cell binding in bioinformatic assessment	Wiggenhorn et al., 2023
EGFR Decoy	N/A	C^*^VRAC^*^_D_(C^*^ARVC^*^)	EGF receptor peptides as decoys for EGF and TGFα	Cardó-Vila et al., 2010
EGF-A	TEX-S2_03	GTNEC(Acm)1LQNPC^*^1 RLMPC^*^2KNGGC(Acm)SHVC ^*^1NDLKIGYEC^*^2L	Inhibition of PCSK9 130-fold over parent EGF-A binder in hypercholesteriomemia	Tombling et al., 2021
IGF-1		C^*^YAAPLKPAKSC^*^	Neuritogenesis and neurogenesis in otic vesicle explants to form cochleovestibular ganglion	Camarero et al., 2003
			Inhibition of Purkinje neural cell viability in developing cerebellum	Croci et al., 2011
TGFα	N-pheTGF-α[21-32]	FC^*^RFLVQEDKPAC^*^	Induced DNA synthesis in NR6/HER fibroblasts	Chamberlin et al., 1995
	h_C_-pep2 h_CC_-pep1	S^#^HFNEYE^#^ C^*^SHFNDYC^*^	Peptide binding to monoclonal antibody for TGFα	Hahn et al., 2001
TGFβ1	pm26TGF-β1	ACESPLKRQCGGGS	Decrease in TNFα and increase in IL10 in T-cell differentiation, and decrease in leukocyte rolling/neutrophil migration	Vaz et al., 2015
TGFβ1 Inhibitor	P17 P144	KRIWFIPRSSWYERA TSLDASIIWAMMQN	TGFβ1 blocking of MV1Lu proliferation and anti-fibrosis in rat liver CCl4 model	Ezquerro et al., 2003
			TGFβ1 inhibition and decreased fibrosis in scleroderma	Santiago et al., 2005
			TGFβ1 inhibition associated with collagen type1 mRNA	Dotor et al., 2007
			TGFβ1 blocking and myocardial fibrosis in hypertension	Hermida et al., 2009
			TGFβ1 blocking—anti-inflammatory and redox balance in noise insult	Murillo-Cuesta et al., 2015
			TGFβ1 blocking from polymer hybrid proteins inhibit SMAD phosphorylation in SNU449 cells	Hanafy et al., 2020

Acronym nomenclature: SNU449, Carcinoma hepatocellular epithelial cell line; CSF1, colony stimulating factor 1; CCl4, chemokine ligand; EGFR, epidermal growth factor receptor; EGF-A, epidermal growth-like domain A; NR6, fibroblast growth factor indicator cell line; IGF-1, insulin-like growth factor 1; IL10, interleukin 10; mRNA, messenger ribose nucleic acid; MV1Lu, mink-derived lung epithelial cell line; PCSK9, proprotein convertase subtilisin/kexin 9; SMAD, small mother's against decapentaplegic subfamily of proteins; TGFα, transforming growth factor alpha; TGFβ1, transforming growth factor beta 1; TNFα, tumor necrosis alpha.

Peptide notations: ^*^nCylic disphulide bridges where n denotes the bridge location for multiple linkages and n is not denoted when only one bridge. _D_ denotes the D stereogenic representation of the subsequent amino acid. Acetamidomethyl (Acm) is a protecting group for controlling the type of disulphide linkage can occur on thiol-containing amino acids, such as C. pE refers to N-pyroglutamylation and # denotes a cyclic bridge through the N-atom of S and C^δ^-atom of E.

Epidermal growth factor (EGF) receptor pathways are highly upregulated after neuronal injury, activating astrocytes (Liu and Neufeld, 2007) and chemotactically mobilizing microglia into lesion sites (Nolte et al., 1997). Cardó-Vila et al. (2010) designed a decoy replicating binding features of the EGF receptor as an EGF pathway inhibitor, while Tombling et al. used the EGF-like domain of the low-density lipoprotein receptor to engineered a peptide therapeutic inhibitor of proprotein convertase subtilisin/kexin type 9 (PCSK9) (Tombling et al., 2021), which could mediate levels of phosphorylated NFκB in reactive astrocytes and microglia (O'Connell and Lohoff, 2020).

IFG-1 has a complex pleiotropic role in neuroinflammation, and is secreted by microglia for various neurotropic and immunomodulatory roles (Myhre et al., 2019). Microglia and astrocytes exposed to high levels of IFG-1 have neuroprotective, inhibit BBB permeability, phagocytic reactivity, and aging-related neurodegeneration (Labandeira-Garcia et al., 2017; Pinto-Benito et al., 2022). Several groups have developed peptides to emulate the IFG-1 binding function (Cascieri et al., 1989; Chrudinová et al., 2018). One cyclic peptide (CYAAPLKPAKSC) has been used successfully to suppress proliferation in prostate carcinoma lines and as a standard agonist for IFG-1 (Bonnefont et al., 2007). Camarero et al. used this peptide to promote neurogenesis and neuritogenesis in the cochleovestibular ganglion, while Croci et al. were able to prolong the survival of Purkinje neurons in the developing cerebellum (Camarero et al., 2003; Croci et al., 2011). Considering this peptide was treated with complex mixtures of CNS cells, it is possible that a pleiotropic response was initiated by neuroglia and could be a potent tool in unlocking their beneficial roles.

## 5 Peptides in glial biology

As microglia are unique phenotypes of neural and immune cells, a handful of peptides are known to be characterized as direct ligands for microglia and astrocytes (Table 4). The ocellatin peptides [K1(1–16) and K1(1–21)] were derived from the amphibian *Leptodactylus vastus* for their antioxidant properties (Sousa et al., 2020). When tested with primary microglia, these helical peptides attenuated ROS, nitrites, and neuroinflammation related to TLR4 and the production of NFκB. One of the more specific microglial-focused peptides was discovered by Kurinami et al. (2016), who developed microglial healing peptides (MHP) based on receptor activator NFκB ligand (RANKL) and its target receptor (RANK). The RANK/RANKL system is a known microglial mediator for ischemic brain injury by inhibiting TLR4 (Iadecola and Anrather, 2011; Shimamura et al., 2014). One major drawback of using RANKL as a direct therapeutic is the induction of osteoclast differentiation (Ferrari-Lacraz and Ferrari, 2011) and eventual bone loss (Tomimori et al., 2009), therefore Kurinami sought to design as a peptide analog that could inhibit TLR4 without the potential osteoporotic complication. To this end, MHP1 inhibited RANKL osteoclast differentiation while attenuating neuroinflammatory behavior and ischemic injury in primary mixed glia-neuronal culture with LPS-induced neuronal death and with an intracerebroventricular peptide injection after a transient middle cerebral artery occlusion.

**Table 4:** Peptides for direct glial targeting.

**Type**	**Name**	**Sequence/label**	**Study**	**References**
Ocellatin	K1 (1–16) K1 (1–21)	GVVDILKGAAKDLAGH GVVDILKGAAKDLAGH LASKV	Anti-inflammation—LPS-TLR4 by ROS and NFκB in CHME3 MG	Sousa et al., 2020
RANKL	Microglial healing peptide MHP1	LMVYVVKTSIKIPS SHNLMKGGSTKNWSGN	Inhibition of LPS TLR4-induced inflammation in ischemia stroke mouse model	Kurinami et al., 2016
ApoE	ApoE_133 − 149_	LRVRLASHLRKLRKRLL	ApoE suppression of TNFα and nitrite in MG	Laskowitz et al., 2001
	EP, ApoE_141 − 149_	LRKLRKRLL	Anti-inflammation—TLR4 and JNK, while activating ERK in MG	Pocivavsek et al., 2009
	ApoE_141 − 149_	(LRKLRKRLL)n, *n* = 2	ApoE peptide dimer stimulated sAPPα and attenuated Aβ	Minami et al., 2010

Acronym nomenclature: Aβ, Amyloid beta; ApoE, apolipoprotein E; JNK, c-Jun N-terminal kinase; CHME3/HMC3, human micoglial cell line 3; LPS, lipopolysaccharides; MG, microglia; MHP1, microglial healing peptide 1; BV2, murine microglia cell line; NFκB, nuclear factor kappa B; RANKL, receptor activator of NF-kB ligand; ROS, reactive oxygen species; sAPPα, stimulating secreted amyloid precursor protein alpha; TLR4, toll-like receptor 4.

Although it may be an unexpected strategy to prime microglia to particular pathogens, it is essential in developing immunotherapeutics. Apolipoproteins are involved in Aβ fibrils and AD, but several peptides have been noteworthy in antimicrobial anti-infective studies (Kelly et al., 2010). Comparable ApoE suppression of TNFα and nitrite in primary murine microglia activation has also been noted with peptides (Laskowitz et al., 2001). Pocivavsek et al. (2009) used the ApoE_141 − 149_ peptide EP to attenuate TLR4 activation, inhibiting the c-Jun N-terminal kinase (JNK) pathway while activating ERK. Dimer form ApoE peptide was the most significant in stimulating secreted amyloid precursor protein alpha (sAPPα) and the microglia-attenuation of Aβ plaques, *in vivo* (Minami et al., 2010).

## 6 Inflammatory peptide candidates in glial mediation of neural pathologies

### 6.1 Cytokine analog peptides

Several peptides were developed with immune targets in mind (Table 5), although their effect on microglia and astrocytes has witnessed minimal evaluation. As microglia bear macrophage/monocyte phenotypes and express most of the same receptors and pathways as many other immune cells, inflammatory-mediating peptides could make excellent candidates to explore in neuroinflammatory systems. Growth factors, neuropeptides, and the cytokine families of interleukins and TNFα are some of the most potent inflammatory mediators that have inspired many peptides and peptide inhibitors. TNFα is one of the most potent pro-inflammatory cytokines in apoptosis and necrosis and has been a primary target for inflammatory mediation in cancer and rheumatic systemic diseases (Idriss and Naismith, 2000). TNFα receptors are prevalent in both microglia and astrocytes. There has been a focus on generating monoclonal antibody inhibitors (Chang et al., 2007; Qin et al., 2007), and several have been clinically evaluated (Benucci et al., 2012), but a demand for more cost-effective peptide variants led to several unique peptides. Chirinos-Rojas et al. first observed an anti-TNFα peptide SEP-7, whose activity could be mediated by retaining 1-3 arginine-based protection groups 4-methoxy-2,3,6-trimethylbenzenesulphonyl (Mtr). They later used an alternative peptide to attenuate cytotoxicity in fibroblasts (Chirinos-Rojas et al., 1997, 1998). Guo et al. (2002) generated a family of cyclic TNFα-targeting peptides, of which binding can be tuned by amino acid amphilicity proximal to the C-terminus. Qin et al. (2006) generated a TNFα antagonist peptide that could competitively inhibit antibody binding and prevent cytotoxicity in L929 cells. Brunetti et al. (2014) synthesized a tri-lysine tetramer peptide and assessed significant binding with Surface Plasmon Resonance and were able to target TNFα receptors in melanoma cells. Sclavons et al. (2013) generated cyclic peptide variants to target TNFα, one of which had specificity to liver tissue in their murine hepatitis model.

**Table 5:** Peripheral peptide candidates for glia in neuroinflammation.

**Type**	**Name**	**Sequence/label**	**Study**	**References**
SOCS-JAK2 Targeting	Tkip	WLVFFVIFYFFR	Inhibition of JAK-STAT pathway in inflammatory disease	Flowers et al., 2004
	SOCS1-KIR	DTHFRTFRSHSDYRRI	In autoimmune encephalitis	Waiboci et al., 2007
	KIRESS	LKTFSSKSEYQLVVNAVRKLQESG	In triple. negative breast cancer	La Manna et al., 2018b
	PS-5	DTC(Acm)RQTFRSH	In type-1 skin, cardiovascular diseases	Doti et al., 2012
			Phosphorylation and cytokine impairment in keratinocytes and T lymphocytes *in vitro* and with an IFNγ treat human skin explants	Madonna et al., 2013
	Linear PS5 Nal1 Cyclic PS5 Cyclic PS5 Nal1	AcDTC(Acm)RQTNalRSH AcC^*^GDTC(Acm)RQTFRSHC^*^G AcC^*^GDTC(Acm)RQTNalRSHC^*^G	In type-1 skin, cardiovascular diseases	La Manna et al., 2017
			Anti-inflammation via JAK/STAT1 and cytokine expression	La Manna et al., 2021
	Cyclic PS5 Na1 peptidomimetic	Ac-c[D**X**^**3**^C(Acm)R**X**^**1**^TNa(1')**X**^**2**^K]H Ac-c[**Y**^**1**^TC(Acm)RQTNa(1')R**Y**^**2**^]H	Use of unnatural building blocks to produce a low-micromolar JAK/STAT1 inhibition	La Manna et al., 2022b
APN/CD13	cyc-LHSPW	C^*^LHSPWC^*^	Inhibition of APN in neuroendocrine prostate cancer	Joshi et al., 2017
	NGR	C^*^NGRC^*^K(Cy5.5)	Optical imaging of HT-1080 and MCF-7 cell lines	von Wallbrunn et al., 2008
		C5.5-22 and C5.5-23	NGR-based non-peptides C5.5-22 and C5.5-23 optimized as CD13 binding peptide NGR benzoyl-aminoproline derivatives using PEG substitutions at the benzoyl moeity	Hahnenkamp et al., 2013
		C^*^NGRC^*^-*yCD* + *5FC*	yCD pro-drug conversion of 5CF to 5-FU targeting CD13 over-expressed cancer cells	Li et al., 2016
	PEP20, PEP24, and PEP173	GYPAY, GFPAY, and GYPAVYLF	CD13 binding ligand designed through Knob-Socket protein packing model	Uddin et al., 2019
IFNγ antagonist		AYC(Acm)RDGKIGPPKLDIRKEEKQI	Inhibition of human IFNγ in Colo 205 cells	Seelig et al., 1995
	Peptide 1-2	_D_(RYT^+^VELA)	Select analog inhibition of ROCK2 and JNK pathway, but not NFκB	Geranurimi et al., 2020
IL2 inhibitor	Cyclotide Kalata B1 [T20K]	GLPVC^*^1GETC^*^2VGGTC ^*^3NTPGC^*^1TC^*^2 SWPVC^*^3TRN	Attenuates proliferation, IL2 and IL2R expression and subsequent TNFα and IL1β in T-lymphocytes	Gründemann et al., 2013
	MOG3	GLPVC^*^1GETC^*^2VGGTC ^*^3NTPGC^*^1TC^*^2RSPFSRVC ^*^3TRNGLPV	Inhibition lymphocyte proliferation in MS model	Wang et al., 2014
IL8/CXCR2 inhibitor	Antileukinate	Ac-RRWWCR-NH_2_	Suppression of neutrophil infiltration and interstitial edema in wet lung model	Hayashi et al., 2002
			Used to measure eosinophil chemotaxis and reduced fibrous thickening in submucosal airway tissue from chronic airborne allergen model	Fukuno et al., 2003
			Decrease in pancreatic MIP-2 and MPO activity in acute pancreatitis model	Bhatia and Hegde, 2007
IL10	IL10NM25	NMLRDLRDAFSRVKTFFQMKDQLDN	IL10Ra binding and anti-mitosis to B cells and lymphoblastoid line	Chang et al., 2020
IL10 inhibitor	P1 and P2	FFKKFFKKFFKKFFKK FFRRFFRRFFRRFFRR	Inhibited growth of IL10 dependant mast cells and LPS mediated-secretion from macrophages P2 promotes IL12 in PBMCs and CD8 T cell response	Ni et al., 2016
IL15 inhibitor	([K6T]P8	KVTAMTC^*^FLL	Inhibition of IL-15Rα and IL6 in a prostate cell line	Santos et al., 2008
TNFα inhibitor	SEP-7	PIR(Mtr)R(Mtr)SDSX	TNFα binding and inhibition mediated by Mtr side chain of R residues	Chirinos-Rojas et al., 1997
	N/A	DFLPHYKNTSLGHRP	TNFα binding and human/mouse inhibition of TNFα cytoxicity in fibroblast cells	Chirinos-Rojas et al., 1998
	N/A	CALWHWWHC C(T/S)WLHWWAC	Direct TNFα binding	Guo et al., 2002
	Z12-TNF-α	SSYPGYSQPTRSYPSS	TNFα antagonist peptide against monoclonal antibody Mab Z12 and caspase activation/cytotoxicity in murine fibroblasts	Qin et al., 2006
	C2-R3 C30-R3	C^*^GSKTQAPC^*^ C^*^PATLTSLC^*^	TNFα binding and staining of concanavalin-treated mouse hepatitis inflammation	Sclavons et al., 2013
	N/A	HIHDDLLRYYGW (KKK tetramer)	TNFα binding to melanoma cells	Brunetti et al., 2014
CHGA	Chromofugin CHR: CHGA47-66	RILSILRHQNLLKELQDLAL	Regulation of alternatively activated macrophages for Inflammatory bowel disease	Eissa et al., 2017
ANXA1	MC-12	QAW	NFκB inhibition for colon cancer	Zhang et al., 2010
		Ac-QAW	NFκB inhibition for colitis	Ouyang et al., 2012
		G^*^1RC^*^2TQAWPPIC^*^2FPD^*^1	Inhibition of NFκB expression Inflammatory bowel disease	Cobos Caceres et al., 2017
PepT1 Substrate	N/A	KVP	Anti-inflammatory cytokine secretion - NFκB and MAP kinase in intestinal epithelial and T cells	Dalmasso et al., 2008
PepT1 Substrate—Soy-Derived	N/A	VPY	Anti-inflammatory cytokines in Caco-2 cells (IL8 and TNFα) rodent colitis (TNFα, IFNγ, IL1β, IL6 and IL17)	Kovacs-Nolan et al., 2012
Gluten Hydrolysate-Derived	N/A	PyroEL	Anti-inflammation (histology) and normalized population of Bacteroidetes and Firmicutes in colitis-induced mice	Wada et al., 2013

Acronym nomenclature: 5-CF, 5-fluorocytosine; 5-FU, 5-fluorouracil; APN, aminopeptidase N; ANXA1, annexin A1; CHGA, chromogranin-A; CHR, chromofugin; CD8, cluster differentiation 8; CD13, cluster differentiation 8; Caco-2, colon-derive epithelial cell line; Colo 205, colon ascitic fluid-derive epithelial cell line; CXCR2, C-X-C chemokine receptor type 2; Cy5.5, cyanine 5.5; yCD, cytosine deaminase; HT-1080, fibrosarcoma-derived epithelial cell line; IFNγ, interferon gamma; IL1β, interleukin 1 beta; IL2, interleukin 2; IL2R, interleukin 2 receptor; IL6, interleukin 6; IL8, interleukin 8; IL10, interleukin 10; IL10Ra, interleukin 10 receptor subunit alpha; IL15, interleukin 15; IL-15Rα, interleukin 15 receptor subunit alpha; IL17, interleukin 17; JAK, Janus kinase; JAK2, Janus kinase 2; KIR, kinase inhibitory region; MIP-2, macrophage inflammatory protein 2; MCF-7, mammary-derived epithelial cell line; A375, melanoma-derived cell line; MAP, mitogen-activated protein; Mab Z12, monoclonal antibody Z12; MS, multiple sclerosis; MOG3, myelin oligodendrocyte glycoprotein epitope 3; MPO, myeloperoxidase; NFκB, nuclear factor kappa B; PBMC, peripheral blood mononuclear cell; PEG, polyethylene glycol; ROCK2, Rho/Rho-associated coiled-coil containing protein kinase 2; SOCS, suppressor of cytokine signaling; SOCS1, suppressor of cytokine signaling 1; STAT, signal transducer and activator of transcription; TNFα, tumor necrosis factor alpha.

Peptide notations: ^*^nCylic disphulide bridges where n denotes the bridge location for multiple linkages and n is not denoted when one bridge. _D_ denotes the D stereogenic representation of the subsequent amino acid. NH_2_ is an amidation on the C-terminus, while Ac refers to the acylation of the N-terminus. ^+^ is an α-amino-γ-lactam (Agl) group. Mtr refers to the protecting group 4-methoxy-2,3,6-trimethylbenzenesulphonyl typically on R residues. The KKK tetramer terms a branched peptides that is built on the side amides and N-terminus of the peptides. Acetamidomethyl (Acm) is a protecting group for controlling the type of disulphide linkage can occur on thiol-containing amino acids, such as C. [Nal(1')] is then non-natural _L_-1-naphthylalanine for higher binding affinity to JAC2. Pyro refers to a pyrrole-group capping the n-terminus. The K(Cy5.5) is a K residue with a cyanine 5.5 attached to the amide side chain.

IL1β is one of the more potent pro-inflammatory cytokines. It has a trophic, proliferative, and reactive effect on microglia (Monif et al., 2016). It has been shown to improve neuronal survival in the retinal system (Todd et al., 2019), but it can compromise and result in cell death for astrocytes (Kralingen et al., 2013). A tripeptide IL1β analog _D_KPT was used by Ferreira et al. (1988) to antagonize IL1β-invoked hyperalgesia in their rat model. Several specialized peptides were designed by Geranurimi et al. (2020) with α-lactam substitutions for D-threonine. Diverse amine and triazole substituents were fabricated to refine the allosteric modulation of the IL1 receptor and tested with *in vitro* assays (retinal microglia) and preterm birth/oxygen-induced retinopathy models. Peptides were found to inhibit Rho/Rho-associated coiled-coil-containing protein kinase 2 (ROCK2) and JNK pathways while ineffective at activating the expected NFκB system.

IL2 is often the focus of T-regulatory cell mediation in neuroinflammatory disease, where the BBB is breached to allow the general immune system to become involved (Yshii et al., 2022). IL2 does share the identical receptor subunits as IL15 and has a pro-inflammatory effect on microglia (nitrites), as opposed to IL15's attenuating effect (Hanisch, 2002). Gründemann et al. (2013) found that the Kalata B1-based cyclotide, a unique peptide that forms intramolecular cyclic bridges across three locations, was capable of inhibiting T-lymphocyte proliferation and down-regulating both the expression of IL2, its receptor, and the subsequent maintenance of baseline TNFα and IL1β. Wang et al. (2014) modified this cyclotide with myelin oligodendrocyte glycoprotein (MOG) epitopes (MOG3) and were able to observe significant myelin remediation in their MS mouse model. The Kalata B1 cyclotide was evaluated to have poor BBB permeability (Melander et al., 2023), yet the BBB is damaged in the late stages of MS to allow encephalitogenic T-cell infiltration. Microglia are known to collaborate with these T-cells (Dong and Yong, 2019) and could have a singular response to cyclotides worth utilizing in earlier disease-stage therapeutics. Although these IL2-mediating peptides might require T-cell intervention to have a more significant effect, IL2 treatments are beneficial in targeting activated astrocytes and Aβ fibrils (Alves et al., 2017), which could be beneficial in treatments for AD.

IL8, also known as the C-X-C chemokine (CXC), has a core role in neutrophil recruitment/degranulation and chemotaxis at core inflammatory insults and infections but can be secreted by many cells with TLRs (Harada et al., 2014), which include microglia and astrocytes (Ehrlich et al., 1998; Robinson et al., 2020). CXC and their receptors have a core role in microglial activation in the NLRP3 inflammasome pathway, as noted in stroke (Werner et al., 2020) and perinatal brain injury (Serdar et al., 2020). One IL8 inhibitor, hexapeptide Antileukinate, has shown promising results in mediating inflammation in lung injury (Hayashi et al., 2002) and acute pancreatitis (Bhatia and Hegde, 2007). Hayashi et al. reduced neutrophil mobilization, significantly reducing interstitial lung fluid buildup in their rodent model. Fukuno et al. (2003) used this peptide and noted a reduction in eosinophil chemotactically-mediated thickening of submucosal tissue in a chronic antigen-exposed rodent model. Bhatia and Hegde noted a decrease in pancreatic myeloperoxidase (MPO) and macrophage inflammatory protein-2 (MIP-2) associated with acute pancreatic inflammation. Antileukinate may have some benefit in treating acute brain insults such as stroke and traumatic brain injury, especially when BBB breach and immune cell infiltration may be a concern.

IL10 is a pleiotropic cytokine that has a primary role in regulating inflammation and is considered one of the most potent 'shut off' mechanisms for restoring microglia homeostasis after a variety of pro-inflammatory insults (Lobo-Silva et al., 2016; Shemer et al., 2020). Unsurprisingly, peptide analogs have been in high demand, and several sequences have emerged recently. Ni et al. generated IL10 inhibitor peptides (P1 and P2) to promote chronic inflammatory resolution using helical repeating amphiphilic residues (Ni et al., 2016). These were tested with T cells and monocytes (U-937 and primary human) and found to enhance IL12 and papillomavirus 16 CD8+ mediated immunity. Chang et al. used *in silico* modeling to generate one candidate IL10 analog (IL10NM25) (Chang et al., 2020), which selectively bound to IL10Ra and reduced the proliferation of monocyte and lymphoblastoid cells. Computational/machine learning approaches by Nagpal et al. (2017) and Singh et al. (2021) have led to hundreds of peptides emulating IL10, which, to date, are untested in cells and tissues.

IL15 is an essential component of the inflammatory signaling cascade, being a pro-inflammatory cytokine regulating homeostasis following insult to the CNS. It is critical in scar tissue formation with microglial accumulation and secretion of IL1β and TNFα, as well as astrocyte production of GFAP (Gomez-Nicola et al., 2010; Shi et al., 2020). As these microglia-astrocyte crosstalks are core to gliosis in every neuroinflammatory pathology, an IL15 peptide analog could be a potent therapeutic tool. The IL15 sequence was derived (Santos et al., 2008) using peptide spot synthesis to target the IL15 receptor alpha (**IL15Rα**). This peptide was evaluated for IL15 activity in two cell lines and was able to attenuate IL15Rα in a human prostate carcinoma cell line. The free cysteine was used to promote a dimer structure, which significantly improved the bioactivity of the peptide. It should be noted that IL15 binding epitopes, as part of antibody structures, have shown promising results in rheumatoid arthritic (Baslund et al., 2005) and psoriatic therapeutic development (Villadsen et al., 2003); therefore, specific molecular machinery may need to be considered in the peptide design for interleukin-based peptides.

### 6.2 Suppressor of cytokine singnalling 2/Janus kinas 2 pathway

Flowers et al. (2004) generated a peptide Tkip to target the autophosphorylation site on Janus kinase 2 (JAK2), which retained some homology to the suppressor of cytokine signaling 2 (SOCS2). The JAK family is a known entity with a crucial role in signaling IFNγ receptors, and JAK2 is specific to IFNγ (Kotenko and Pestka, 2000), which is a potent driver in TLR activation and switch for the adaptive immune response over the innate microglial response (Kann et al., 2022). Comparable to JAK2, Flowers et al. found that Tkip inhibits IFNγ's antiviral behavior and promotes an adaptive presentation response associated with major histocompatible complex class I (MHCI**)** (Flowers et al., 2004). Another peptide associated with the kinase inhibitory region (KIR) for binding to JAK2, named SOCS1-KIR, was evaluated by Waiboci et al. (2007). After confirming binding to pJAK2 (1001–1013), the SOCS1-KIR peptide functioned as an agonist to IFNγ and blocked SOCS1-induced inhibition of signal transducer and activator of transcription 3 (STAT3) phosphorylation, suggesting its use as a SOCS-1 antagonist. Such potent microglial neuro-inflammatory mediating peptides could lead to treatments for oncological and immunological disorders, which are often associated with uncontrollable tyrosine kinase activity (Blume-Jensen and Hunter, 2001; Tsygankov, 2003). Specific to the CNS, the JAK2/SOCS could be a core target in mediating cancer and neurodegenerative disorder (Nicolas et al., 2013) and has been associated with microglia and astrocytes in neurological disorders comparably to other components of the immune system (Jain et al., 2021).

One of the more prevalent families of JAK2-targeting peptides was discovered and characterized in D. Marasco's group. Initially discovered in a peptide library engineered to screen comparable KIR peptides by Doti et al. (2012), a new combinatorial peptide PS-5 was found with binding constant values in the nanomolar range able to reduce the phosphorylation of STAT1 and expression of IRF-1 in keratinocytes. Madonna et al. (2013) corroborated PS-5's JAK2 impairment in keratinocytes and T lymphocytes *in vitro* and with an IFNγ treatment of human skin explants. La Manna et al. evaluated PS-5's ability to mimic SOCS-1 in vascular smooth muscle cells (VSMCs). They could derive cyclic and non-natural naphthyl (Nal1) containing variants with better stability (La Manna et al., 2017). They employed a surface plasmon resonance technique to characterize their peptide binding with a more robust quality. La Manna et al. (2021) used the cyclic forms PS-5 to inhibit the oxidative stress associated with JAK-mediated tyrosine phosphorylation and pro-inflammatory cytokine expression in VSCMs. La Manna's team was also able to generate SOCS3 mimetic peptides (KIRESS) to inhibit the cytokine production of triple-negative breast cancer subtypes and their subsequent tumor growth and pulmonary metastasis in murine xenografts (La Manna et al., 2018b). La Manna et al. (2022b) performed a systematic *ad-hoc* assessment of several cyclic lactam bridge KIR-SOCS1 peptide modifications to refine micromolar affinity toward JAK2. Modifications included subtle alterations in ring stability/flexibility with citrulline (Cit), ornithine (Orn), and diaminopimelic acid (Dap) substitutions to generate several inhibitor variants. La Manna et al. have compiled more comprehensive reviews on anti-inflammatory peptides involved in the JAK2/SOCS system (La Manna et al., 2018a, 2022a). Despite their wide success, these KIR peptides have yet to be applied to CNS-related neuroinflammatory mediation or tumor suppression.

Considering its potent pro-inflammatory effect on microglia and astrocytes, there has been interest in generating IFNγ antagonist peptide analogs (Seelig et al., 1995). In microglia, IFNγ antagonist has been linked to inhibiting neuroinflammation associated with AD and PD, as well as being clinically relevant to the pathophysiology of schizophrenia (Kato et al., 2007). IFNγ antagonism in astrocytes has a protective mediation of autoimmune-related neurological disability (Hindinger et al., 2012). It should be noted that autoimmune antagonism has been shown to have opposing effects in microglia and astrocytes signaling (Ding et al., 2015), so some degree of specificity in targeting glia might be required to produce the most beneficial response, which complex peptide ligands could produce.

### 6.3 Aminopetidase N/cluster differentiation 13 pathway

Metalloproteinase peptide cleavage is critical in activating peptide fragments in various inflammatory and wound-healing events. The M1 aminopeptidase N (APN) or CD13 cleavage event promotes viral activation (Chen et al., 2012) cancer angiogenesis and metastasis (Zhang and Xu, 2008; Wickström et al., 2011) making it a noteworthy target for the mediation of corona viral inflammation (Ruan et al., 2023) and anti-cancer, some of which reached clinical evaluation (Xu et al., 2011; Jiang et al., 2016). Microglia produce higher levels of APN than peripheral monocytes and macrophages when assessing enkephalin inactivation (Lucius et al., 1995), associated with pain sensation and mood disorders (Henry et al., 2017). Furthermore, APN is a known regulator for astrocyte-microglial crosstalk in the brain renin-angiotensin pathway during neuroinflammation, which is crucial in regulating CNS blood pressure (Kim et al., 2022). Joshi et al. (2017) generated a selective APN-inhibitor peptide (cyclic HSPW) through structural profiling and X-ray crystallographic assessment. They demonstrated the peptide's inhibitory and anti-tumorgenic activity in PC3/DU145 cells and DU145 grafted mice. Although one application was tested, the peptide inhibition of this system could lead to analgesics, mood stabilizers, and neuroinflammation/blood pressure attenuating peptides if focused toward CNS glia.

The APN-targeting NGR peptide sequence has limited investigation as an immune mediating peptide but has led to several advances in binding affinity refinement (Xu and Li, 2005). Cyanine 5.5 (Cy5.5) imaging in cancer-based cell lines HT-1080 and MCF-7 was performed by von Wallbrunn et al. (2008), while Hahnenkamp et al. substituted poly ethylene glycol (PEG) spacers on benzoyl moieties of NGR derivatives to improve fluorescent targeting (Hahnenkamp et al., 2013). Li et al. (2016) added an anti-tumor pro-drug property with a cytosine deaminase chimeric peptide capable of converting 5-fluorocytosine (5-CF) to a cytotoxic 5-fluorouracil (5-FU) at the CD13 of cells receptor modeled across a broad spectrum of cell lines (A375, A431, MCF-7, HT-1080, MDA-MB231, MDA-MB468). Uddin et al. (2019) introduced a Knob-Socket protein packing modeling system to generate two 5-mer and one 8-mer peptide (PEP20, 24, and 173) with an order of magnitude higher binding affinity over the cyclic NGR control. Although the NGR peptide may have little effect on APN inhibition compared to other targets, it certainly provides a strong candidate for shuttling other effective ligands to APN-expressing glia.

### 6.4 Intestinal marcrophage-mediating peptides

Several small inflammatory regulating peptides, some as small as three amino acids, have been discovered to mitigate bowel inflammation through intestinal macrophages. Wada et al. (2013) used a gluten hydrolysate-derived pyroEL peptide in an acute DSS-based colitis model as an anti-inflammatory/antimicrobial agent. Kovacs-Nolan et al. (2012) treated their DSS-colitis model with the soy-derived VPY in DSS-induced rodent colitis. They noted a significant reduction in pro-inflammatory cytokines: TNFα, IFNγ, IL1β, IL6, and IL17. The tripeptide KVP was used by Dalmasso et al. (2008) to inhibit pro-inflammatory cytokine secretion through the NFκB and mitogen-activated protein (MAP) kinase pathway in intestinal epithelial and T cells and eventually attenuate dextran sulfate sodium (DSS)/2,4,6-trinitrobenzene sulfonic acid (TNBS)-induced colitis in mice.

Several groups derived small peptides from Annexing A1 (ANXA1), a potent mediator of glucocorticoid anti-inflammation through NFκB. The NFκB/MAP pathways are crucial for induction and maintenance in the CNS (Mendonca et al., 2018). They are hallmarks of microglial activation in AD-related Aβ plaque-induced inflammation (Tilstra et al., 2011). Zhang et al. (2010) used the ANXA1 tripeptide QAW in their intestinal cancer xenograft model and reduced tumor tissue growth by 58%. The ANXA1-derived peptide MC-12 was used by Ouyang et al. (2012) as a NFκB inhibitor in mice with DSS-induced colitis and were able to restore cytokines TNFα, IFNγ, and IL1β, IL6, and IL10 to a baseline. Cobos Caceres et al. (2017) grafted a cyclic form of MC-12 into a sunflower trypsin inhibitor scaffold. The cyclic MC-12 peptide significantly improved over the linear MC-12 in reducing the inflammation in their acute colitis model in mice while maintaining stability in plasma.

Neuropeptide derivatives have been used in a handful of studies. Bettenworth et al. (2011) used an α-MSH C-terminus fragment tripeptide K(d)PT in their in *vitro/in vivo* IL10 deficient colitis mice models to note a reduced severity in inflammation and improved transepithelial electrical resistance after IFNγ and TNFα activation. As noted previously, α-MSH peptides also have a profound effect on microglia through the inhibition of TLR2 and TLR4 (Carniglia et al., 2016) and the attenuation of Aβ activation (nitric oxides, TNFα, and IL6) (Galimberti et al., 1999; Lindberg et al., 2005). Akgül et al. (2006) noted a decrease in rodent NFκB and pancreatic inflammation when they treated their rat model with their TNBS acid-induced colitis with octreotide, a somatostatin derivative. Also mentioned previously, somatostatins have a potent effect on microglia, including the inhibition of IL3-stimulated proliferation, tyrosine phosphorylation, and anti-amyloidosis in AD (Feindt et al., 1998; Tundo et al., 2012).

Chromogranin-A (CHGA) is a vesicle-based acidic protein and peptide precursor in various immune regulating functions (Chanat and Huttner, 1991; D'amico et al., 2014). One CHGA-derived peptide, Chromofungin (CHR: CHGA_47 − 66_), is a promising candidate that is involved in antimicrobial (Yoo, 1992; Maget-Dana, 1999; Lugardon et al., 2001) and inflammatory regulation (Ghia et al., 2004; Metz-Boutigue et al., 2010). Eissa et al. (2017) used the CHR peptide to activate gut macrophages into an alternative phenotype capable of attenuating oxidative stress and pro-inflammatory cytokine release, reducing colitis in a mouse model. Microglia bear many phenotypical similarities and have a fundamental crosstalk role with intestinal macrophages (Verheijden et al., 2015). CHGA is considered to promote a neurotoxic phenotype in brain microglia (Ciesielski-Treska et al., 1998), but this has also been shown to promote a phenotypical switch (Taupenot et al., 1996), leading to a neuronal-apoptotic response (Ulrich et al., 2002). CHGA is also a hallmark of neurodegenerative cerebral spinal fluid (Kaiserova et al., 2021) and tissue lesions (van Luijn et al., 2016). It may be the apoptotic outcome over a necrotic cascade that allows the favorable colitis remediation seen by Eissa et al., but this has yet to be verified with CHR-treated microglia.

## 7 Innovative tools and techniques in the discovery of neuroglial specific peptides

With a few exceptions, most of the peptides discussed were found to be targets for many other cell types or known receptors. Using naïve approaches from library techniques, few efforts have been made to discover peptides specific to glia or mechanisms of glia with a focus on microglia and/or astrocytes. The major benefit to employing such tools allows for the discovery of new mechanisms focused on cellular or bio molecular targets. In particular, phage display has yielded the most significant results, which utilizes a peptide-coat expressing bacterial virus with extensive sequence diversity (<billion sequences). The M13 filamentous phage has been a popular choice due to its non-essential regions for modification. Despite their prevalence use across many types of cells and tissue, only a few studies have utilized M13 or other filamentous phage for microglial and astrocyte panning (Table 6). Examples include the identification of a select peptide (SFTYWTN) from a family ^S^/_T_F^T^/_X_YW consensus site from E20 microglia (Samoylova et al., 2002) and a small number of binders to primary murine microglia using fd phage (Lundin et al., 2003). Terashima et al. (2018) employed a combination of M13 phage display for isolating peptides specific to M1 and M2 microglia phenotypes. Koss et al. (2019, 2023) observed changes in binding patterns of 58 candidate peptides to primary rat microglia across various phenotypes using M13 phage display. Functional analysis suggested roles in synaptic pruning, neuroprotection, and neurodegenerative diseases. Additionally, Zhou et al. used a C7C phage system to discover a heptapeptide against Aβ42 that reduced reactive oxygen species and microgliosis *in vivo*.

**Table 6:** Glial-specific phage-derived peptides.

**Name**	**Sequence**	**Study**	**Glial phenotype**	**References**
SFTYWTN	SFTYWTN	One sequence from a family with a ^S^/_T_ F^T^/_X_ YW motif to bind to EOC 20 MG using M13 phage	Ramified	Samoylova et al., 2002
II-4:25 II-3:5 II-3:19 V-1:19 V-2B:9 V-2B:20	IGRLNVFAMTGVRGR ASRSVLSGR WCLVDVPELGAYWRLACTR LGFSYWETPNEGVLI NGMVGGCFG ALENEDGLRIAMGRL	Peptide clones compared against immune cells using fd phage	Ramified	Lundin et al., 2003
BMHP1	SKPPGTSS	Viability of NR/RG/ACs using M13 phage	N/A	Nowakowski et al., 2004; Koutsopoulos and Zhang, 2013
Gas6 MFG-E8	Full-length proteins	Phage-mediated cellular uptake applying Gas6 and MFG-E8 eat-me pathway in BV-2 MG using T7 phage	Phagocytic	Caberoy et al., 2009
Tubby	Full-length protein	Tubby-mediated uptake in microglial MerTK receptor using T7 phage	Phagocytic	Caberoy et al., 2010a,b, 2012
ZW1	ZW1SMSARQL	Aβ42 fibrils target with reactive BV-2 MG effect, using M13 phage	Aβ42 activated	Zhou et al., 2014
AS1 MG1 MG2	CLNSSQPSC CHHSSSARC CNTGSPYEC	3 peptides identified for ACs and MG using M13 phage	M1 and M2	Terashima et al., 2018
Various	58 SX7 peptides	Peptides across phenotypes of ACs and MG using M13 phage	Ramified, LPS activated, and primed	Koss et al., 2019, 2023

Acronym nomenclature: Aβ42, Amyloid beta 42; AC, astrocyte; BMHP1, bone marrow homing peptide 1; fd, *Fd* specific filamentous phage; M13, *Ff* filamentous phage; Gas6, growth arrest—specific 6; T7, icosahedral-capsule phage; LPS, lipopolysaccharide; MG, microglia; EOC, murine microglial cell line; M1/M2, macrophage polar phenotypes reactive/ramified; MFG-E8, milk fat globule-EGF factor 8 protein; NR, neuron; BV-2, murine microglial cell line; RG, radial glia.

The bone marrow homing peptide 1 (BMHP1) was discovered against hematopoietic stem cells (Nowakowski et al., 2004). Bjornson et al. (1999) demonstrated shared differentiation and adhesion pathways between stem cells of neural and bone marrow origins, making these motifs attractive in the context of neural tissue engineering. SKPPGTSS is also involved in the family of neuronal apoptosis inhibitor proteins (Deveraux and Reed, 1999). BMHP1 was also eventually used to differentiate neural precursors into astrocytes in a 3D culture environment (Gelain et al., 2006) and investigated the viability of neural and glial cells (radial glia/astrocytes) within peptide hydrogel scaffolds. The highest percentage of living cells in the hydrogels containing the SKPPGTSS peptide remained after 3 months, indicating that this motif is essential in supporting neuronal cell viability (Koutsopoulos and Zhang, 2013).

The T7 phage system is emerging as the preferred method due to several advantages over filamentous phage. These include faster bacterial growth, direct insertion of large cDNA libraries into the T7 genome for capsid fusion, broader compatibility with various libraries, simpler and more efficient affinity elution, and enhanced viability during recombination (Deng et al., 2018). To date, Caberoy et al. (2009, 2010a, 2012) have conducted a series of T7 studies focused on microglial phagocytosis, identifying peptides that mediate the engulfment of debris through the c-mer proto-oncogene tyrosine kinase (MerTK) receptor and “eat-me” signals. Notably, only one of these studies directly employed primary microglia and BV-2 cells for panning (Caberoy et al., 2009), while the rest used full-length protein display to facilitate a phagocytic response.

Despite the limited peptide discovery studies available, the peptides have yielded a treasure of new information. With clever experimental design, these display tools can be expanded into other pathologies and classes of neuroglia. Further, glial are anatomically disctinct across regions of the CNS (Tan et al., 2020) and mammalian physiologies (Toledano Furman et al., 2020), which could also be explored. New molecular features are being added to the phage themselves to tailor phage toward unique targets or applications; cyclic and glycopeptides (Ng et al., 2012; Ng and Derda, 2016), chelates (Wright and Deonarain, 2007), and BBB traffickers (Majerova et al., 2014; Urich et al., 2015), which could be pivotal in challenging *in vivo* phage display techniques. New mRNA display technique are immerging with sequence diversities reaching the trillions (Newton et al., 2020), yet these require transfection, which is more successful with astroglia than microglia. Recently, functionally microglia have been discovered in cerebral organoids (Ormel et al., 2018), which are excellent for modeling genetic disorders from patient-derived induced pluripotent stem cells. Considering all of the tools emerging in the peptide-display fields, many exciting projects and discoveries await the glial researcher.

## 8 Conclusions and future directions

At a cursory glance, there may be limited options for choosing methods for the immune and bioengineering of immune cells, microglia, and support cells astrocytes, which are crucial in the homeostasis and maintenance of neural health and neuroinflammatory mediation. However, many peptides have been characterized in the distant and recent literature. Neuropeptides and hormonal peptides are powerful tools for the control of inflammation, neuroprotection, and gliotransmission. However, they are also potent ligands, albeit highly specific, for receptors in many cells across several mammalian systems, making them useful in specific cases (isolated cell culture studies, *ex vivo* treatments, and drug delivery systems). Despite neuroglia's role in ECM reconstruction and growth factor secretion in wound healing and pathophysiology, a handful of peptides have been discovered to replicate these features effectively. Peptides are known to be excellent candidates in replicating cytokine analogs, targeting cytokine receptors, and regulating immune and inflammatory responses from macrophages and monocytes in other systems. With further exploration, these could lead to many potent therapeutics, as these pathways are also known in microglia and astrocytes. Specificity to neuroglia remains elusive for many of these peptides. However, recent biopanning techniques, such as phage display, have uncovered many peptides capable of distinguishing glia from other cell types. Further, these peptides can identify sub-phenotypes of glia, including M1 and M2, or activated and ramified, which is of great interest in screening and immunotechnologies (blotting, microscopy, and cytometry). The specificity of such peptides may not confer a biological response, and their target and origin proteins are often difficult to elucidate. Nevertheless, these options allow for the exciting and pleiotropic design of chimeric peptides and bioconjugates that could unlock meaningful tools and therapeutics for the glial researcher.

## Author contributions

BB: Conceptualization, Visualization, Writing – original draft, Writing – review & editing. KK: Conceptualization, Visualization, Writing – original draft, Writing – review & editing, Funding acquisition, Supervision.

## Glossary

**Table:**

α-MSH	Alpha-melanocyte stimulating hormone
3-NP	1-Nitropropionic acid
5-CF	5-fluorocytosine
5-FU	5-fluorouracil
Aβ /Aβ42	Amyloid beta / amyloid beta 42
AC	Astrocytes
ACTH	Adrenocorticotropin or adrenocorticotropic hormone
AD	Alzheimer's disease
ADNP	Activity dependent neuroprotector protein
AG	Acyl-Ghrelin
ALS	Amyotrophic lateral sclerosis
AM	Adrenomedullin
AM2	Intermedin
AM2R	Adrenomedullin 2 receptor
AMYR1-3	Amylin receptor 1-3
ANXA1	Annexin A1
APN	Aminopeptidase N
APEL	Apelin-13
APJ	Apelin
ApoE	Apolipoprotein E
BB	Bombesin peptides
BBB	Blood brain barrier
BBR1-3	Bombesin receptors 1-3
BDNF	Brain-derived neurotrophic factor
BMHP1	Bone marrow homing peptide 1
B2R	Type 2 bradykinin receptor
cAMP	Cyclic adenosine mono phosphate
CCK-8	Cholecystokinin / octeotride
CCKBR	Cholecystokinin B receptor
CCKR	Cholecystokinin receptor
CCl4	Chemokine ligand 4
CD8	Cluster differentiation 8
CD11b	Cluster differentiation 11b
CD13	Cluster differentiation 13
CD40	Cluster differentiation 40
CD86	Cluster differentiation 86
CD200	Cluster differentiation 200
cDNA	Complementary deoxyribosenucleic acid
CGRP	Calcitonin gene-related peptide
CGRPR	Calcitonin gene-related peptide receptor
CHGA	Chromogranin-A
CHR	Chromofungin
CNS	Central nervous system
COX2	Cyclooxygenase 2
CRF	Corticotrophin releasing factor
CRF1-2	Corticotropin releasing factor type 1-2 receptor
CSF1	Colony stimulating factor 1
CST-14	Cortistatin 14
CST-17	Cortistatin 17
CXC	C-X-C chemokine
DSS	Dextran sulfate sodium
DOP	δ Opioid receptor
EAE	Experimental autoimmune encephalomyelitis
ECM	Extracellular matrix
EGF	Epidermal growth factor
EGF-A	Epidermal growth-like domain A
ERK	Extracellular signal regulated kinase
Ex-4	Exendin-4
FGF	Fibroblast growth factor
FGFR	Fibroblast growth factor receptor
FGL	Fibroblast growth loop
FRM	Fibroblast growth factor receptor activation motif
GALA-2-11	Galanin
GALR1-3	Galanin receptor 3
Gas6	Growth arrest—specific 6
GC-C	Guanylate cyclase C
GFAP	Glial fibrillary acidic protein
GHS	Growth hormone secretogues
GHSR1	Growth hormone secretagogue receptor
GHRHR	Growth-hormone-releasing hormone receptor
GLP-2	Glucagon-like peptide
GLP1R-2R	Glucagon-like peptide-1-2 receptor
GPR39	G-protein coupled receptor 39
GM-CSF	Granulocyte-macrophage colony-stimulating factor
GUCY2CR	Guanylyl cyclase 2C receptors
GRF	Growth hormone releasing factor
GRP	Gastrin release peptide
GRPR	Gastrin-releasing peptide receptor
HD	Huntington's disease
HK-1	Hemokinin
Iba1	Ionized calcium binding adaptor molecule 1
IDE	Insulin-degrading enzyme
IFNγ	Interferon gamma
IGF1	Insulin-like growth factor-1
IKKβ	IκB kinase β
IL1	Interleukin 1
IL1β	Interleukin 1 beta
IL2	Interleukin 2
IL2R	Interleukin 2 recptor
IL3	Interleukin 3
IL4	Interleukin 4
IL6	Interleukin 4
IL8	Interleukin 8
IL10	Interleukin 10
IL10NM25	Interleukin 10 analog
IL10R	Interleukin 10 receptor
IL10Ra	Interleukin 10 receptor alpha
IL11	Interleukin 11 receptor
IL15	Interleukin 15
IL15Ra	Interleukin 15 receptor alpha
IL17	Interleukin 17
iPSC	Induced pluripotent stem cell
JAK2	Janus kinase 2
JNK	c-Jun N-terminal kinase
KIR	Kinase inhibitory region
KOP	κ Opioid receptor
LENK	Leu-enkephalin
LEP	Leptin peptide
LEPR/OBR	Leptin/obesity receptor
LEPRa/b	Leptin receptor short/long fragments
LPS	Lipopolysaccharide
M1/M2	Macrophage polar phenotypes reactive/ramified
M15	Galantide
Mab Z12	Monoclonal antibody Z12
MAP	Mitogen-activated protein
MAPK	Mitogen-activated protein kinase
MC1R	Melanocortin 1 receptor
MC4R	Melanocortin 4 receptor
MENK	Met-enkephalin
MerTK	c-Mer proto-oncogene tyrosine kinase
MFG-E8	Milk fat globule-EGF factor 8 protein
MG	Microglia
MHCI	Major histocompatibility complex I
MHCII	Major histocompatibility complex II
MHP	Microglial healing peptide
MIP-2	Macrophage inflammatory protein-2
MOG	Myelin oligodendrocyte glycoprotein
MOP	μ Opioid receptor
MPO	Myeloperoxidase
mRNA	Messenger rubosenucleic acid
MS	Multiple sclerosis
Mtr	4-methoxy-2,3,6-trimethylbenzenesulphonyl
NADPH	Nicotinamide adenine dinucleotide phosphate
Nal1	Non-natural naphthyl
NCAM	Neural cell adhesion molecule
NR	Neuron
NFκB	Nuclear factor kappa B
NK1R-3R	Neurokinin-1-3 receptor
NKA	Neurokinin A
NKB	Neurokinin B
NLRP3	Nod-like receptor protein 3
NMB	Neuromedin B
NMBR	Neuromedin B receptor
NMDA	N-methyl-D-aspartate
NMC	Neuromedin C
NMU	Neuromedin U
NMUR1-2	Neuromedin U receptors type 1-2
N/OFQ	Nociceptin/Orphanin FQ
NOP	Nociceptin receptor
NPY	Neuropeptide Y
NPYR1-5	Neuropeptide Y receptor type 1-5
NSC	Neural stem cell
OG	Oligodendrocyte
OP	Opioid receptor
OXT	Oxytocin
OXTR	Oxytocin 1 receptor
PAC	Pituitary adenylate cyclase-activating polypeptide receptor
PACAP-27	Pituitary adenylate cyclase-activating polypeptide 27
PACAP-38	Pituitary adenylate cyclase-activating polypeptide 38
PBMC	peripheral blood mononuclear cell
PCSK9	Proprotein convertase subtilisin/kexin type 9
PD	Parkinson's disease
PDYN	Prodynorphins
PEG	polyethylene glycol
PENK	Proenkaphalin
PI-3	phosphatidylinositol-3 kinase
pJAK2	Phospho-Janus kinase 2
PNOC	Prepronociceptin
POMC	proopiomelanocortin
PP	Pancreatic polypeptide
UROC	Urocorting
RAMP	Receptor-activity modifying proteins
RANK	Receptor activator NFκB ligand receptor
RANKL	Receptor activator NFκB ligand
RG	Radial glia
ROCK2	Rho/Rho-associated coiled-coil containing protein kinase 2
ROS	Reactive oxygen species
sAPPα	Secreted amyloid precursor protein alpha
SCI	Spinal cord injury
SOCS2	Suppressor of cytokine signaling 2
SP	Substance P
SMAD	Small mother's against decapentaplegic subfamily of proteins
SST-14	Somatostatin peptide 14
SST-28	Somatostatin peptide 28
SSTR1-5	Somatostatin receptors 1-5
STAT3	Signal transducer and activator of transcription 3
TBI	Traumatic brain injury
TGF-α	Transforming growth factor alpha
TGF-β	Transforming growth factor beta
TLR2	Toll-like receptor 2
TLR4	Toll-like receptor 4
TNBS	2,4,6-trinitrobenzene sulfonic acid
TNFα	Tumor necrosis factor alpha
TP5	Thymopentin
UROC	Urocortin I
VDP	Vitronectin-derived peptide
V1aR	Vasopressin 1a receptor
VIP	Vasoactive intestinal peptide
VPAC1-2	Vasoactive intestinal polypeptide receptor 1-2
VPN/AVP	Vassopressin/arginine vasopressin
VSMC	Vascular smooth muscle cell
VEGF	Vascular endothelial growth factor
